# Small molecule AX-024 reduces T cell proliferation independently of CD3ϵ/Nck1 interaction, which is governed by a domain swap in the Nck1-SH3.1 domain

**DOI:** 10.1074/jbc.RA120.012788

**Published:** 2020-04-21

**Authors:** Kirsten Richter, Arne C. Rufer, Magali Muller, Dominique Burger, Fabio Casagrande, Tabea Grossenbacher, Sylwia Huber, Melanie N. Hug, Philipp Koldewey, Andrea D'Osualdo, Daniel Schlatter, Theodor Stoll, Markus G. Rudolph

**Affiliations:** ‡ I2O Disease Translational Area, pRED Pharma Research and Early Development, Roche Innovation Center Basel, F. Hoffmann-La Roche Ltd., Grenzacherstrasse 124, 4070 Basel, Switzerland; § Therapeutic Modalities, Lead Discovery and Medicinal Chemistry, pRED Pharma Research and Early Development, Roche Innovation Center Basel, F. Hoffmann-La Roche Ltd., Grenzacherstrasse 124, 4070 Basel, Switzerland

**Keywords:** adaptor protein, crystal structure, T-cell receptor (TCR), protein drug interaction, cell proliferation, structure-function, protein structure, nuclear magnetic resonance (NMR), analytical ultracentrifugation, surface plasmon resonance (SPR)

## Abstract

Activation of the T cell receptor (TCR) results in binding of the adapter protein Nck (noncatalytic region of tyrosine kinase) to the CD3ϵ subunit of the TCR. The interaction was suggested to be important for the amplification of TCR signals and is governed by a proline-rich sequence (PRS) in CD3ϵ that binds to the first Src homology 3 (SH3) domain of Nck (Nck-SH3.1). Inhibition of this protein/protein interaction ameliorated inflammatory symptoms in mouse models of multiple sclerosis, psoriasis, and asthma. A small molecule, AX-024, was reported to inhibit the Nck/CD3ϵ interaction by physically binding to the Nck1-SH3.1 domain, suggesting a route to develop an inhibitor of the Nck1/CD3ϵ interaction for modulating TCR activity in autoimmune and inflammatory diseases. We show here that AX-024 reduces T cell proliferation upon weak TCR stimulation but does not significantly affect phosphorylation of Zap70 (ζ chain of T cell receptor–associated protein kinase 70). We also find that AX-024 is likely not involved in modulating the Nck/TCR interaction but probably has other targets in T cells. An array of biophysical techniques did not detect a direct interaction between AX-024 and Nck-SH3.1 *in vitro*. Crystal structures of the Nck-SH3.1 domain revealed its binding mode to the PRS in CD3ϵ. The SH3 domain tends to generate homodimers through a domain swap. Domain swaps observed previously in other SH3 domains indicate a general propensity of this protein fold to exchange structural elements. The swapped form of Nck-SH3.1 is unable to bind CD3ϵ, possibly representing an inactive form of Nck in cells.

## Introduction

**Figure 1.**
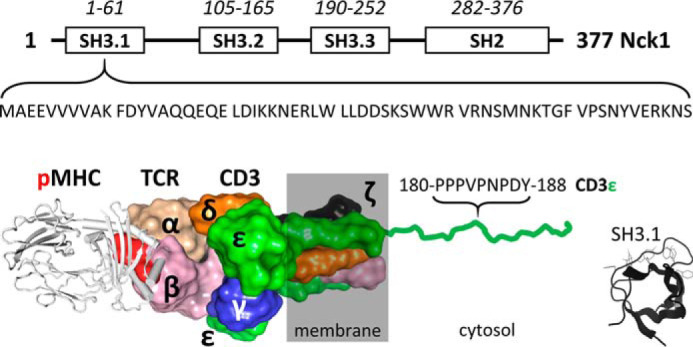
**Human Nck1 domain organization and interaction of its N-terminal SH3.1 domain with the CD3ϵ subunit of the T cell receptor.** *Top*, sequence boundaries of the three SH3 domains and the C-terminal SH2 domain of Nck. The sequence of the SH3.1 domain interacting with CD3ϵ is noted. *Bottom*, sketch of the peptide-MHC/TCR interaction (based on a superposition of PDB entries 2CKB and 6JXR). The TCR and CD3 subunits are labeled. Cytosolic tails are omitted for clarity except for one of the CD3ϵ subunits to highlight the PRS that binds to Nck1-SH3.1. Tyr-188 in the PRS (*red*, *boldface type*) is the first tyrosine in the ITAM of CD3ϵ. The Nck1/CD3ϵ interaction can be mimicked *in vitro* by the isolated Nck1-SH3.1 domain (residue range 4–59 is sufficient; *inset*) and CD3ϵ-derived peptides.

As part of the adaptive immune response, T cells help protect an organism from microbial invasion and transformed cells by differentiating between self-antigens and foreign antigens. Peptide antigens bound to the major histocompatibility complex (MHC)^4^ are scanned by the heterodimeric αβT cell receptor (TCR). During negative selection in the thymus, TCRs specific for self-peptides are normally deleted. These usually interact weakly with peptide-MHC complexes. The αβTCR includes the heterodimeric CD3γϵ and CD3δϵ complexes and the homodimeric CD3ζζ (Fig. 1). The ITAMs (immunoreceptor tyrosine-based activation motifs) in the cytoplasmic parts of CD3ζζ are phosphorylated by the Src-type kinases Fyn and Lck (1). These phosphotyrosine side chains are recognized by the tyrosine kinase Zap70 (ζ-chain–associated protein of 70 kDa), which in turn phosphorylates and activates LAT (linker for activation of T cells) and Slp76 (SH2 domain-containing leukocyte protein of 76 kDa). Other proteins essential for actin reorganization, including the cytosolic adapter proteins WASP (Wiskott–Aldrich syndrome protein) and Nck (noncatalytic region of tyrosine kinase), are then recruited (1, 2) to initiate formation of branched actin filaments.

Nck proteins are composed of three N-terminal SH3 domains and a single C-terminal SH2 domain (reviewed in Ref. 3; Fig. 1). Humans have two similar Nck isoforms, Nck1 and Nck2, which share 69% sequence identity (80% similarity) and seem to have overlapping functions (3, 4), although separate activities begin to emerge (5). Whereas in mice, knockout of either *nck* gene has no apparent phenotype, a double knockout is lethal (6). Usually, the Nck-SH2 domain binds to phosphorylated tyrosine residues in receptor tyrosine kinases, including epidermal growth factor receptor, platelet-derived growth factor, and Ephrin receptor. The SH3 domains then recruit proteins containing PRS to form larger complexes. By interacting with, among other proteins, WASP, WIP (WASP-interacting protein), and the p21-activated kinase PAK 1, receptor-induced signals are relayed to changes in the actin cytoskeleton.

Nck amplifies weak antigen signals and initiates signal transduction predominantly via Lck-mediated phosphorylation and Zap70 activation (7, 8). Nck recruitment is required for complete T cell activation, and its inhibition dampens TCR signaling by reducing Zap70 phosphorylation (9). Nck was reported as more important in amplifying T cell signaling in response to weak (prototypic self-antigens) than strong (potentially pathogen-derived) antigens. Thus, blocking the Nck/TCR interaction appears as a route for the treatment of autoimmune diseases, including lupus erythematosus, psoriasis, asthma, multiple sclerosis, and transplant rejection, while at the same time avoiding dampening activation of pathogen- and tumor-specific T cells (2, 10).

The objective of the present study was to initiate development of an inhibitor of the Nck/TCR interaction, specifically by blocking binding of the first SH3 domain in Nck (Nck1-SH3.1) to the PRS in the CD3ϵ subunit of the TCR. The starting point was the exciting observation of a small molecule, termed AX-024, to inhibit T cell proliferation specifically in response to weak antigens (10). Surface plasmon resonance (SPR) and NMR experiments *in vitro* appeared to support physical interaction of AX-024 with the Nck1-SH3.1 domain, leading to its assignment as a potential inhibitor of the Nck/CD3ϵ interaction (10). We sought to follow this rationale and started out by studying the biological effect of AX-024 on T cells and its interaction with Nck1-SH3.1 *in vitro*. We find that whereas AX-024 does indeed inhibit T cell proliferation, the drug neither substantially inhibits Zap70 phosphorylation in response to weak TCR stimulation nor interacts directly with Nck1-SH3.1. In addition, the reported importance of the Nck/CD3ϵ interaction for T cell signaling could not be confirmed in mutant Jurkat cells where the Nck/CD3ϵ interaction was abrogated. On the other hand, we detected polypharmacology for AX-024, which might explain its activity in reducing T cell proliferation by pathways other than via inhibition of the Nck/CD3ϵ interaction.

During our studies, it became clear that Nck-SH3.1 exhibits conformational diversity and can swap half the number of its residues to form a dimer. Dimer formation is reversible and temperature-dependent. The crystal structures of the Nck1-SH3.1 monomer in complex with a CD3ϵ peptide and of dimers revealed that the dimers are unable to bind to CD3ϵ. Structurally similar dimers, but also others with very different topology, have been described for SH3 domains from a number of proteins. Interestingly, a swapped SH2 domain was described for Nck as well, rendering this adaptor protein one of the few examples to exhibit two different domain swaps within the same molecule. Given the repeated occurrence of domain swaps in SH2/3 domains, the question arises of whether there might be an associated biological function.

## Results

**Figure 2.**
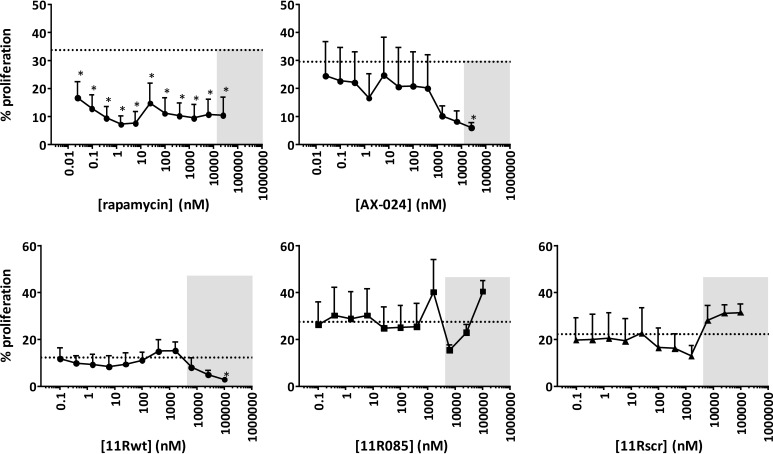
**CD3ϵ-derived peptides do not inhibit T cell proliferation.** CD4 T cell proliferation was assessed upon stimulation with anti-CD3ϵ by CFSE dilution as a measure for cellular proliferation, analyzed on day 4. For rapamycin and AX-024–treated cells, the *dashed line* indicates control proliferation at a DMSO concentration of 0.2%. For CD3ϵ-derived peptides 11Rwt, 11R085, and 11Rscr, the *dashed line* indicates control proliferation at 0.3% DMSO, 0.3% PBS. 11Rwt has sequence R_11_-G_3_-RGYNKERPPPVPNPDY, 11R085 R_11_-G_3_-Q(dK)KECPPPVPKRDY, and 11Rscr R_11_-G_3_-PKVRECPDYK(dK)PQP. A polyarginine tag is used to enable cell penetration of the peptides (10). The *gray boxes* indicate concentrations of compound leading to decreased cell viability. These values should be disregarded. Binding of peptides 11Rwt and 11R085, but not 11Rscr, to Nck-SH3.1 was confirmed by NMR spectroscopy (Fig. S1). *Asterisks*, *p* < 0.05 as determined by a repeated-measure one-way analysis of variance with Holm–Sidak's multiple-comparison test to DMSO/PBS. *Dotted lines*, mean DMSO/PBS values. *Error bars*, S.D.

It has been shown previously (10) that the small molecule AX-024 inhibits proliferation of primary T cells, presumably by physically blocking the interaction of Nck1 and CD3ϵ. Based on this interesting finding, we planned to develop this molecule into a protein-protein inhibitor (PPI) of the Nck1/CD3ϵ interaction, setting out by confirming its mode of action. In a proliferation assay, the biological activity of AX-024 as a CD4 T cell proliferation inhibitor was confirmed (Fig. 2). Pre-experiments established experimental conditions (*i.e.* low anti-CD3 concentrations and absence of co-stimulation) that induce a weak T cell stimulation leading to moderate T cell proliferation (not shown). As expected, AX-024 inhibited T cell proliferation, confirming previous results (10) (Fig. 2). In the next step, CD3ϵ-derived peptides that were reported to compete with the Nck/CD3ϵ interaction in a cellular context and *in vitro* (11) were tested for their T cell–inhibitory effects. These peptides were already characterized by Borroto *et al.* (11). The peptides were rendered cell-penetrating by poly-Arg sequences (12) and shown to be taken up at significant levels starting from >10 μm (11). Peptides 11Rwt and 11R085 (sequences in Fig. S1) contain the canonical recognition sequence for Nck-SH3.1 and should therefore compete for interaction with Nck, abrogating any TCR signaling depending on the Nck/CD3ϵ interaction. As a control, a scrambled peptide (11Rscr) with the same composition as 11R085 but lacking the canonical PRS was used. As expected, using NMR, the peptides 11Rwt and 11R085 indeed bind to Nck1-SH3.1 *in vitro*, whereas 11Rscr does not (Fig. S1). However, none of the peptides inhibited CD4 T cell proliferation at concentrations up to 1 μm (Fig. 2). At concentrations exceeding 6 μm, all peptides were cytotoxic (marked by boxes in Fig. 2) *i.e.* below the concentrations where the peptides entered cells efficiently (10 μm) (11). Similarly, incubation with these peptides did not affect CD8 T cell proliferation (at concentrations low enough to avoid cytotoxic effects; data not shown). Thus, whereas the CD3ϵ-derived peptides do bind specifically to Nck1-SH3.1 *in vitro*, they do not behave as specific T cell proliferation inhibitors in our hands. At this point, we took another step back and asked the more general question of how important the Nck/CD3ϵ interaction is for T cell proliferation.

### The Nck1-SH3.1/CD3ϵ interaction is of limited relevance for TCR signaling

**Figure 3.**
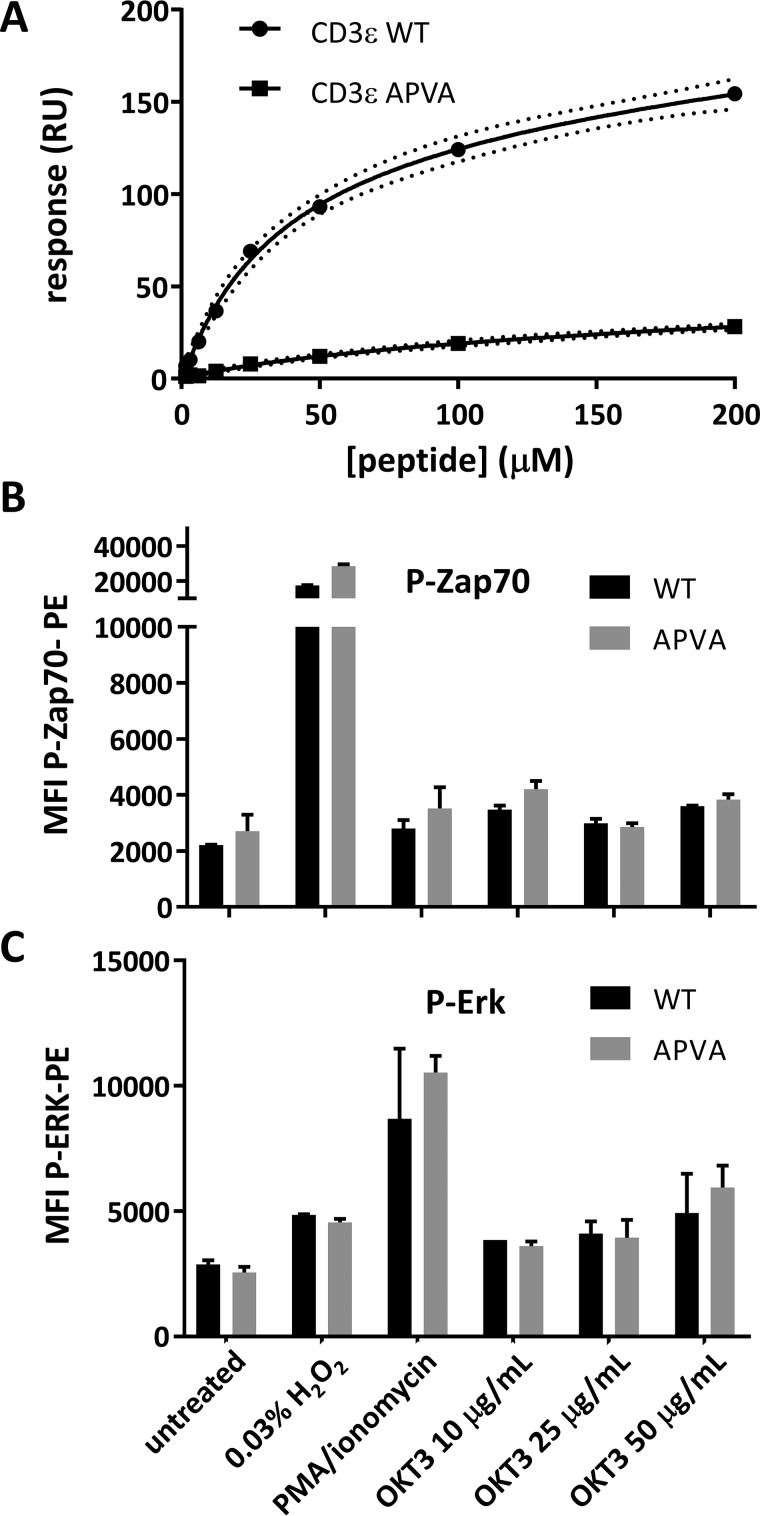
**The APVA mutation abrogates the CD3ϵ/Nck-SH3.1 interaction but does not affect Zap70 and ERK phosphorylation in Jurkat cells.** *A*, SPR confirmed the interaction between Nck-SH3.1 and WT CD3ϵ peptide RGQNKERP**P**PV**P**NPDY. APVA mutant CD3ϵ peptide RGQNKERP**A**PV**A**NPDY (*squares*) binds ∼10-fold less strongly to Nck-SH3.1 than the WT. *B* and *C*, WT or APVA mutant Jurkat cells were either left untreated or treated with H_2_O_2_ or PMA/ionomycin or were stimulated for 5 min with plate-coated anti-CD3 (OKT3). Zap70 (*B*) and ERK (*C*) phosphorylation were then measured by flow cytometry. *Error bars*, S.D.

Previous work demonstrated that a knock-in mouse line with a CD3ϵ variant carrying the sequence APVA instead of the canonical PPVP in the PRS lacks full antigen-induced T cell activation (11). We confirmed by SPR analysis that CD3ϵ peptide variants carrying the APVA sequence do not bind *in vitro* (Fig. 3*A*); hence, this sequence change should be sufficient for abrogation of the Nck/CD3ϵ interaction also in cells. Using CRISPR/Cas9, a mutant Jurkat cell line carrying this variant was generated, providing an *in vitro* tool to study the importance of the Nck/CD3ϵ interaction for T cell signaling. Production of the CD3ϵ APVA variant in the mutant cell line was confirmed by sequencing. Western blotting and FACS were used to choose clones showing a similar CD3ϵ level as nonmanipulated Jurkat cells (not shown). We also confirmed that the APVA mutant cell line proliferated with a rate comparable with WT Jurkat cells (not shown). Unexpectedly, we found that upon weak stimulation in the absence of co-stimulation of WT *versus* APVA mutant Jurkat cells, similar levels of phosphorylated Zap70 (P-Zap70) and P-ERK could be detected (Fig. 3*B*). Therefore, the abrogation of CD3ϵ/Nck1-SH3.1 interaction in cells does not bear functional consequences for TCR signaling in this cell line and under the conditions tested.

### AX-024 does not significantly influence Zap70 phosphorylation

**Figure 4.**
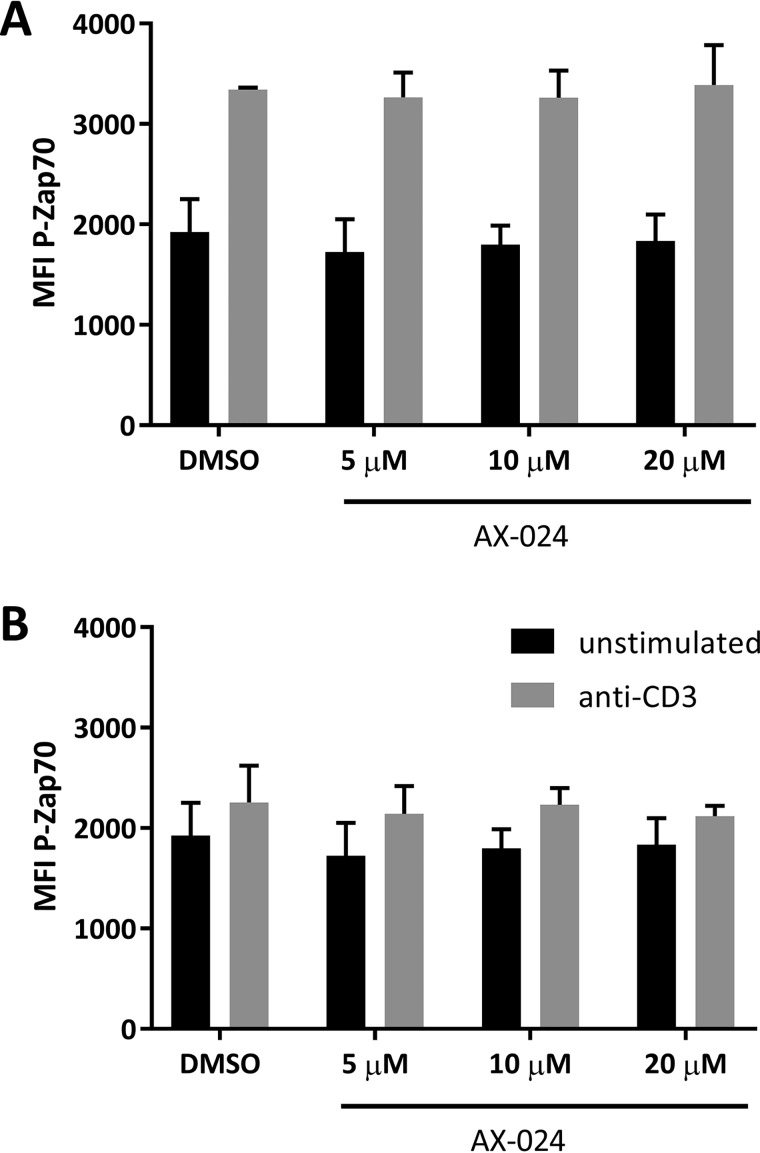
**AX-024 does not significantly reduce Zap70 phosphorylation.** Healthy donor PBMCs were isolated rested overnight and pretreated with 20 μm AX-024 or vehicle prior to stimulation with 20 μg·ml^−1^ (*A*) or 10 μg·ml^−1^ (*B*) anti-CD3. The cells were gated on live cells, CD3, and CD4 for assessment of P-Zap70. *Error bars*, S.D.

It was shown previously that Nck contributes to TCR signaling after weak stimulation of T cells. In accordance with these data, and under the assumption that AX-024 blocks the Nck/CD3ϵ interaction, it was shown that AX-024 reduces Zap70 phosphorylation to the same level as in unstimulated T cells (10). To obtain a strong dependence of TCR signaling on Nck, different stimulation conditions with low concentrations of anti-CD3 in the absence of co-stimulatory signals were assessed in the presence of AX-024 (Fig. 4). However, none of the conditions tested resulted in significant reduction of Zap70 phosphorylation. Different antibody clones together with different strengths and periods of stimulation were also compared, but these experiments did not result in substantial reduction of Zap70 phosphorylation (data not shown).

Whereas our experiments replicate the inhibitory effect of AX-024 on immediate T cell activation, it remains less clear whether a direct interaction of CD3ϵ and Nck is required for T cell proliferation and whether AX-024 acts as a protein-protein interface inhibitor, physically blocking the interaction between Nck and CD3ϵ. As the concept of a PPI for autoimmune and inflammatory indications remains attractive, we analyzed the potential interaction of AX-024 with Nck1-SH3.1 *in vitro*. First, we quantified CD3ϵ peptide binding to Nck1-SH3.1 by SPR, followed by direct and competitive binding studies with AX-024.

### A CD3ϵ-derived peptide binds Nck1-SH3.1 in vitro

**Figure 5.**
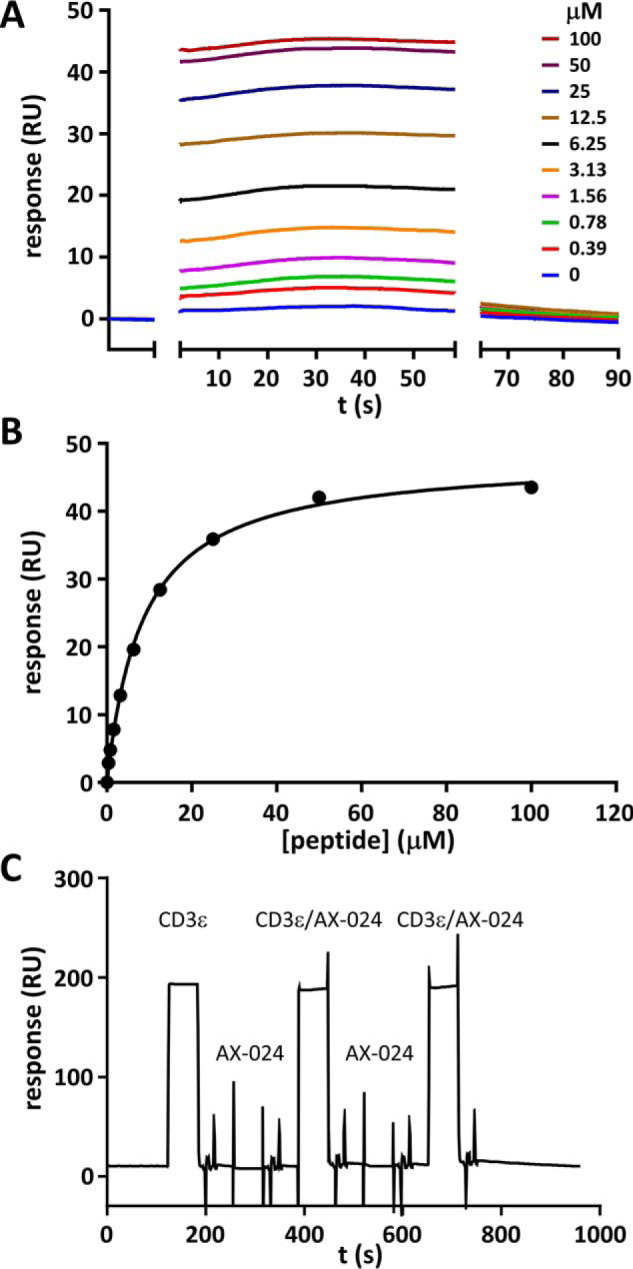
**Detection of CD3ϵ interaction with Nck1-SH3.1 by SPR.** *A*, SPR time traces of the CD3ϵ peptide RGQNKERPPPVPNPDY binding to immobilized Nck1-SH3.1. The association and dissociation phases were completed too quickly on the time scale of the SPR experiments to yield information and were removed for clarity. *B*, titration of the CD3ϵ/Nck1-SH3.1 interaction. The fit to the data yields a *K_d_* value of 8.3 ± 0.1 μm (five replicates, Table S1). *C*, the small molecule AX-024 does not bind to Nck1-SH3.1 in this setup. Five consecutive experiments are shown. From the *left*, the sensorgram shows injection of 100 μm CD3ϵ peptide, followed by a wash step to remove all of the peptide, and then 100 μm AX-024 was injected. After another wash step, a mixture of 100 μm each of CD3ϵ peptide and AX-024 was injected, followed by a wash step and another AX-024 injection at 100 μm. The last injection was again the mixture of AX-024 and CD3ϵ peptide, each at a concentration of 100 μm. Only the presence of CD3ϵ peptide results in a consistent change of the SPR signal, which is unaffected by AX-024. Hence, AX-024 does not bind to Nck1-SH3.1 under these conditions.

Nck1-SH3.1 carrying a biotin moiety was immobilized on a streptavidin-coated SPR sensor chip. A short CD3ϵ-derived peptide encompassing the residue range 173–188 binds to Nck1-SH3.1 in a concentration-dependent manner (Fig. 5*A*). The equilibrium signals allowed determination of the *K_d_* value for this interaction as 8.3 ± 0.1 μm (Fig. 5*B* and Table S1).

The association and dissociation phases of the sensorgrams are completed within a few seconds and are thus too rapid compared with the time resolution of the SPR instrument to allow extraction of quantitative information. Hence, no rate constants could be calculated from the sensorgrams. However, assuming a typical association rate constant for small proteins of 10^6^ m^−1^·s^−1^, the dissociation rate constant *k*_off_ of the CD3ϵ/Nck1-SH3.1 complex should be ∼9 s^−1^. This *k*_off_ is large enough for a competing compound to show an inhibitory effect on the SPR time scale, which was tested next.

Using the same approach as for the peptide, binding of the inhibitor AX-024 to Nck1-SH3.1 was tested. In competition experiments, AX-024 was co-injected with CD3ϵ but failed to show an effect on binding of CD3ϵ to immobilized Nck1-SH3.1 (Fig. 5*C*). Furthermore, no direct binding of AX-024 to immobilized Nck1-SH3.1 was detectable up to a concentration of 100 μm ligand, despite using a sensor surface with higher capacity (see “Experimental procedures”) to increase the expected small SPR signal elicited by a small molecule.

### NMR detects rapid association and dissociation of the Nck1-SH3.1/CD3ϵ complex

**Figure 6.**
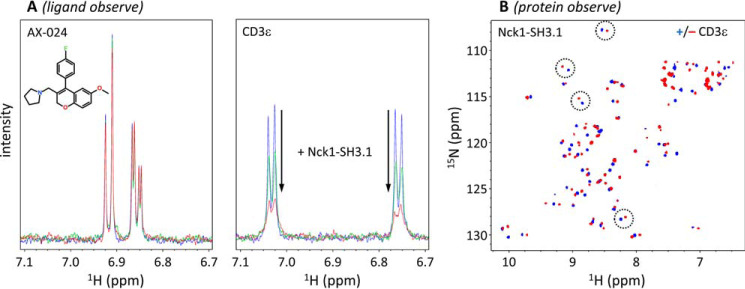
**NMR detects CD3ϵ but not AX-024 binding to Nck1-SH3.1.** *A*, ligand-observed NMR. *Left*, aromatic AX-024 resonance signals (50 μm solution) do not show any spectral changes upon the addition of monomeric (*red*) and dimeric (*green*) Nck1-SH3.1 (10 μm; residues 1–61). *Right*, in contrast, substoichiometric amounts of Nck1-SH3.1 lead to line broadening of CD3ϵ peptide RGQNKERPPPVPNPDY Tyr-188 H_δ_ and H_ϵ_ resonances, indicating fast-exchange binding. The addition of monomeric (*red*) Nck1-SH3.1 shows strong line broadening (*i.e.* binding to the peptide), in contrast to dimeric Nck1-SH3.1 (*green*), which shows much less line broadening. *B*, protein-observed ^1^H-^15^N HSQC NMR confirms Nck1-SH3.1 (residues 4–59) binding to the CD3ϵ peptide by distinct chemical shift perturbations (representative pairs ± CD3ϵ are *circled*), as also reported in Ref. 10. For this experiment, the monomeric form of SH3.1 was used. The *blue* and *red spectra* are without and with CD3ϵ peptide, respectively.

To confirm the data obtained by SPR in solution, the interaction of the same CD3ϵ-derived peptide with Nck1-SH3.1 was studied by ligand-observed NMR (Fig. 6). The addition of monomeric Nck1-SH3.1 (see below for data on the dimeric form) at substoichiometric amounts lead to significant line broadening of tyrosine H_δ_ and H_ϵ_ resonance signals in the CD3ϵ peptide (Fig. 6*A*). This observation is characteristic for fast-exchange ligand binding, as was already detected by SPR. In contrast, AX-024 aromatic signals of the para-substituted fluorobenzyl group remained the same upon titration with Nck1-SH3.1 (Fig. 6*A*), although this group was implicated in SH3.1 binding (10). As a result, although the CD3ϵ peptide does bind to Nck1-SH3.1, no binding of AX-024 was detected by NMR, confirming the SPR results.

To exclude nonspecific interaction of the CD3ϵ peptide with Nck1-SH3.1, 2D protein-observed NMR was performed (Fig. 6*B*). Some Nck1-SH3.1 NH backbone chemical shifts undergo large perturbations upon addition of CD3ϵ peptide. The resulting chemical shift pattern of CD3ϵ peptide-saturated Nck1-SH3.1 shows identical features as published previously (10). In conclusion, whereas the specific binding of CD3ϵ peptides to Nck1-SH3.1 could be quantified, we were unable to detect binding of AX-024 to SH3.1, neither in a cellular context nor *in vitro*. These data also explain why we could not detect an effect of AX-024 on Zap70 phosphorylation (Fig. 4).

### Possible polypharmacology of AX-024

The unexpected observation that AX-024, in our hands, did not bind to Nck1-SH3.1 *in vitro*, but had significant proliferation-inhibitory effect on T cells, prompted us to test the compound for possible binding to other proteins. AX-024 was submitted to a selectivity panel comprising 50 recurring off-targets, including G protein–coupled receptors, channels, transporters, and metabolic enzymes (Eurofins, CEREP). Single-point measurements at 10 μm AX-024 were performed and sorted for >50% inhibition of binding or enzymatic activity (Table S2). A set of six G protein–coupled receptors and a Ca^2+^ channel met the criteria for strong inhibition. Whereas the corresponding genes of the binding partners are not expressed by blood T cells, as judged by mRNA levels, the rather high number of seven “off-targets” in this limited panel indicates likely polypharmacology of AX-024. Due to the absence of gene expression of any of the observed candidates, we did not engage in further target identification of AX-024 in T cells but held on to the concept of a PPI for the CD3ϵ-Nck1 interaction.

In principle, a PPI can be developed using the surface of one of the target proteins alone. A solution NMR structure is available for Nck1-SH3.1 (13), although the precision of NMR-derived coordinates may be suboptimal for drug design. We thus determined crystal structures of Nck1-SH3.1 both alone and in complex with a CD3ϵ-derived peptide to delineate precisely the site of the CD3ϵ-Nck1 interaction.

### Crystal structure of the Nck1-SH3.1 domain and its complex with a CD3ϵ peptide

**Figure 7.**
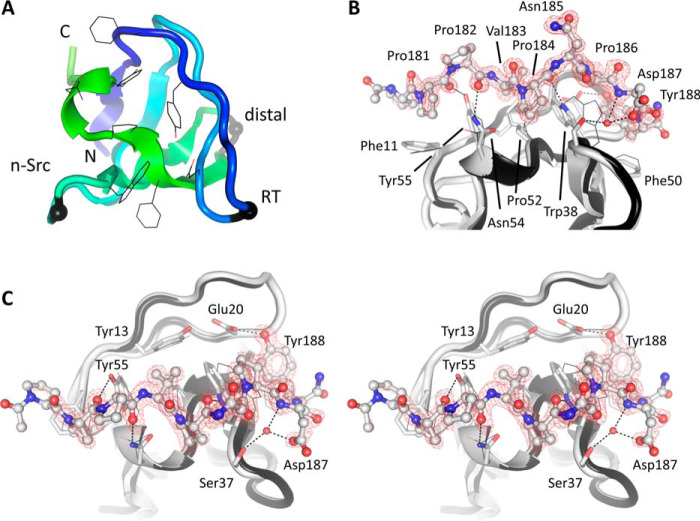
**Crystal structure of the Nck1-SH3.1 domain and its complex with CD3ϵ peptide.** *A*, the apo-structure of Nck1-SH3.1 is shown as a *ribbon* with the peptide-binding side chains drawn as *lines*. Three distinctive loop regions of SH3 domains are indicated by *black spheres. B*, Nck1-SH3.1 in complex with the CD3ϵ peptide PPPVPNPDY. The omit electron density, contoured as a *red mesh* at 3 RMSD, shows that the first and last residues of the peptide are less well-defined than the central part, implying flexibility. *C*, the *cross-eyed stereo view* is rotated by 90° about the *horizontal axis* compared with *B*. The largest structural changes in the Nck1-SH3.1 domain upon peptide binding are side-chain adjustments and inclusion of a water molecule (*red sphere*) that is absent in the apo-Nck1-SH3.1 structure. Atoms between SH3.1 and the peptide in hydrogen-bond geometry are connected by *dashed lines*. Note that a phosphorylated Tyr-188 would not fit into the binding site of the SH3.1 domain.

The apo-form of Nck1-SH3.1 (residues 4–59) was crystallized at a pH of 6.5 (Table S3) in triclinic space group P1 with two molecules in the asymmetric unit. The diffraction data (Table S4) were phased by molecular replacement using the SH3 domain of human hematopoietic cell kinase (HCK) (14). Residues 2–57 are well-defined by electron density, whereas the remaining residues are disordered in this crystal form. As expected, the archetypical SH3 fold of a β-sheet curved into a half-barrel is conserved (Fig. 7*A*). When superimposed on the NMR structure of the Nck1-SH3.1 domain, the root mean square deviation (RMSD) is 1.2 Å for the core SH3 residues 5–57. The side chains interacting with CD3ϵ (*black lines* in Fig. 7*A*) are in similar conformations as in the NMR solution structure (13, 15). Superposition of Nck1-SH3.1 with the crystal structure of the HCK SH3 domain (40%/68% sequence identity/homology), reveals a similar RMSD of 0.8 Å. For comparison, the two protomers of Nck1-SH3.1 in the asymmetric unit superpose with RMSD of 0.24 Å. The largest differences are in the two N-terminal residues, outside the peptide-binding region.

The complex of Nck1-SH3.1 with minimal CD3ϵ peptide ^180^PPPVPNPDY^188^ was crystallized from the same conditions as the apo-form (Table S3) but in orthorhombic space group I2_1_2_1_2_1_ with different packing interactions. The two complexes in the asymmetric unit of this structure closely superimpose with RMSD of 0.24 Å. Slight structural changes compared with the apo-SH3.1 domain are apparent (RMSD 0.45 Å; Fig. 7, *B* and *C*). The most prominent, peptide-induced structural changes pertain to the side chains of SH3.1 residues Phe-11, Trp-38, Phe-50, Pro-52, Asn-54, and Tyr-55. Most of these changes are rather subtle, with side-chain movements between 0.5 and 1.2 Å. An important exception is the indole ring of Trp-38, which swings by ∼3 Å and becomes locked between CD3ϵ side chains Val-183, Pro-184, and Pro-186. The CD3ϵ side chain Pro-181 packs on top of SH3.1 side chain Phe-11, and CD3ϵ Pro-184 wedges between SH3.1 side chains Ser-37 and Asn-54. These two CD3ϵ residues are mutated in the cell lines used to study Nck/CD3ϵ interactions in cells (11) (this study). Although a few hydrophobic interactions will be lost in the mutant, residual affinity of a double-Ala variant might be retained (Fig. 3*A*). Direct hydrogen bonds are formed almost exclusively between side chains of SH3.1 and main-chain atoms of the peptide. A notable exception is CD3ϵ side chain Tyr-188 hydrogen-bonding to Nck1 side chain Glu-20 (Fig. 7*C*). In addition to the hydrogen bond, Tyr-188 also stacks on Phe-50 in Nck1, making this position a selectivity hotspot: Tyr-188 is the first tyrosine in the ITAM of CD3ϵ and needs to be unphosphorylated for Nck1 to bind to CD3ϵ (16). The crystal structure explains this necessity: for steric reasons, phosphorylation of Tyr-188 would abolish all interactions with Glu-20 and Phe-50. A second selectivity-imposing residue is CD3ϵ side chain Val-183. The side chain entertains hydrophobic interactions with SH3.1 side chains Tyr-13, Trp-38, Pro-52, and Tyr-55, which form a cavity that seems tailored for valine. Thus, selectivity of CD3ϵ for Nck-SH3.1 is established mainly by hydrophobic contacts from residues in the SH3.1 domain acting on the CD3ϵ hotspots Val-183, Pro-184, Pro-186, and Tyr-188. Despite this selectivity, CD3ϵ appears to retain significant flexibility when bound to Nck1, as judged from a comparison of the two peptide-SH3.1 complexes in the asymmetric unit. A lateral, ∼0.5-Å shift of the peptides is present, centered at Pro-184, together with alternate conformations of Asp-187 and Tyr-188 in one of the peptide structures (Fig. S2). These conformational changes are in line with the value of 8.3 μm for the equilibrium dissociation constant. Of note, neither the apo-form nor the SH3.1 structure with the peptide removed could serve as frameworks to dock AX-024 in a manner similar to what was proposed earlier (10).

### Unexpected domain swaps in crystal structures of the Nck1-SH3.1 domain

During the crystallization efforts that led to the monomeric apo- and peptide-bound Nck1-SH3.1 crystal structures, seven more structures were determined that all exhibited domain swaps, leading to dimers. The starting material for all crystallization experiments was the monomeric form of Nck1-SH3.1. The dimeric crystal structures were derived from very different precipitants (Table S3), none of which contained particularly harsh conditions, such as extremes of pH or high percentage of organic solvents that are known to trigger domain swaps (17, 18). In addition, different crystals containing monomeric and dimeric Nck1-SH3.1 species were grown from the same conditions (Table S3), indicating that the crystallization conditions did not play a major role in shifting the monomer-dimer equilibrium.

**Figure 8.**
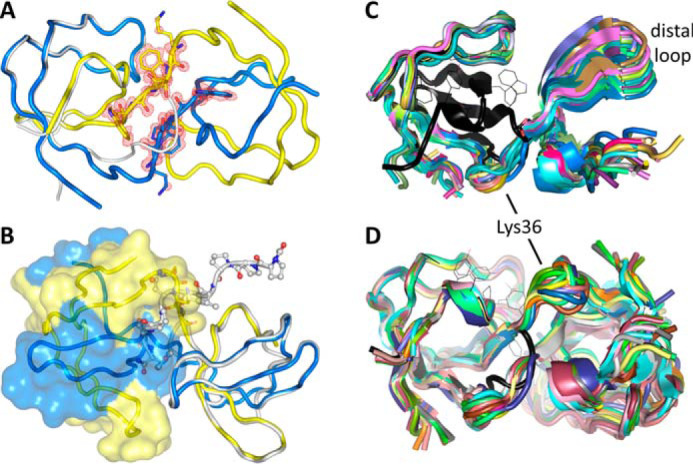
**Domain-swapped Nck1-SH3.1.** *A*, the domain swap at Lys-36 in the n-Src loop. Omit electron density is shown in *red* at the 3 RMSD level for the sequence ^36^KSWW^39^. The main chains follow an anti-parallel β-sheet in the dimer (shown as *yellow* and *blue ribbons*). The monomeric SH3.1 is drawn as a *gray ribbon* for reference. *B*, one half of the swapped dimer is shown as a *transparent surface*. The CD3ϵ peptide-Nck1-SH3.1 complex is superimposed onto the other half of the dimer. The peptide, drawn as a *ball-and-stick model*, clashes with the surface, indicating no or only residual affinity for the complex. *C* and *D*, structural variability of the dimers. The nonswapped, monomeric Nck1-SH3.1 domain is shown in *black* as a reference. *C*, 38 individual Nck1-SH3.1 chains from crystal structures were superimposed on the N-terminal half of the SH3.1 domains, showing the differences for the second half. *D*, superposition of 21 individual dimers onto one of the protomers shows how the structural variability of the domain-swapped chains results in very different conformations of the dimers.

The swaps are located at the exact same position in all structures (*i.e.* Lys-36 in the n-Src loop), clearly visible based on the high-resolution electron density (Fig. 8*A*). Dimers are formed by exchange of the C-terminal 25 residues, almost half of the entire SH3 domain. The interface thus generated contains a short anti-parallel β-sheet encompassing the CD3ϵ sequence ^36^KSWW^39^.

Superposition of the Nck1-SH3.1 structure with the bound CD3ϵ peptide shows that domain swap and tight peptide binding are mutually exclusive (Fig. 8*B*). The C-terminal half of the SH3.1-binding sequence would get into steric conflict with the second SH3.1 domain in the dimer. This hypothesis was tested in solution using NMR on a preparation containing dimeric Nck1-SH3.1, purified by gel permeation chromatography. The ligand-observed NMR experiment with a substoichiometric concentration of CD3ϵ peptide resulted in much smaller line broadening compared with the monomeric Nck1-SH3.1 (Fig. 6*A*, *right*), indicating weak binding. As the crystal structure of the dimer indicates no or very weak binding of peptide to the SH3.1 domain, the NMR result may be due to residual monomer or a weak and nonspecific residual affinity of CD3ϵ for dimeric Nck1-SH3.1. For completeness, AX-024 was tested for binding to the dimeric Nck1-SH3.1, but no interaction was observed (Fig. 6*A*, *left*), excluding the dimer as the target for this compound.

### The domain-swapped Nck1-SH3.1 domain adopts a variety of conformations

In total, there are 41 independent Nck1-SH3.1 chains forming 21 dimers (structure 5QU8 has a single chain in the asymmetric unit, and the symmetric dimer is formed by a crystallographic operation). All of the dimers are swapped in the same manner, but with different juxtapositions of the second SH3.1 domain with respect to the first. To assess the structural variability of the dimers, 38 individual chains with well-defined electron density throughout were superimposed onto the first half of the molecule (Fig. 8*C*). The hinge at Lys-36 provides quite a bit of flexibility that allows the dimer to pack in many different crystal lattices. Indeed, the hinge region varies over ∼6 Å within the superposed ensemble, and the distal loop covers a distance of ∼8 Å in the dimers. Such a wealth of structural data on a single protein is rare, allowing the conclusion that the range of conformations observed in the crystals is likely to also be present in solution if a domain swap occurs in the context of authentic Nck.

Given the large number of swapped, independent chains present in different crystal forms and grown under different conditions, swapping seems an intrinsic property of this SH3 domain. It is unclear whether the domain swap occurred during protein concentration after the last purification step or as part of the phase transition during crystallization. Due to the inherent structural variability of the Nck1-SH3.1 dimer, development of a PPI appears more difficult than anticipated. We therefore addressed the question of what might have triggered the domain swap and how stable these species are in solution.

### Reversible formation of stable monomers and dimers

**Figure 9.**
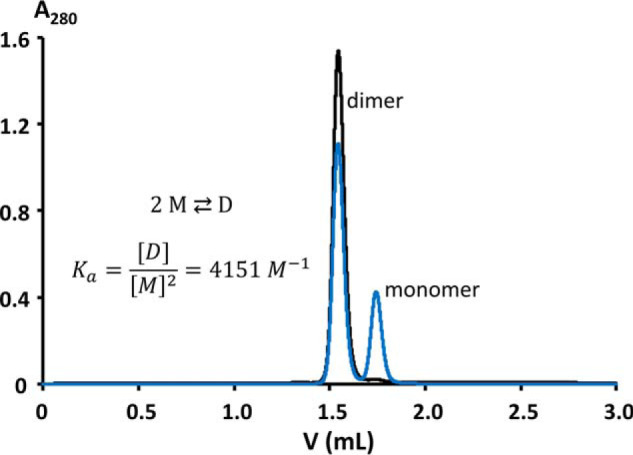
**Gel-permeation chromatography detects a stable dimer of Nck1-SH3.1.** 5 μl of a 1.14 mm solution of Nck1-SH3.1 residues 4–59 were injected onto a Superdex 75 Increase (3.2/300 mm, *V*_tot_ = 3 ml, 0.1 ml·min^−1^ flow, exclusion volume 0.8 ml; GE Healthcare), equilibrated in 25 mm HEPES/NaOH, pH 7.8, 150 mm NaCl. The *black chromatogram* reports on pure dimer, whereas the *blue chromatogram* was obtained after overnight incubation of the dimer sample at 37 °C. The integrated dimer signal is 1527.2 milli-absorbance units (mAU). This signal is reduced to 1111.4 mAU for the heated sample. The monomer generated accrues 424.7 mAU, or 27.6% of the total absorption. Assuming equilibrium and no further monomer/dimer exchange during chromatography and sample cooling to room temperature, the concentration of monomer [*M*] is 315 μm. Applying double the extinction coefficient for the dimer compared with the monomer, [*D*] is 412 μm. The association equilibrium constant is calculated to be 4151 m^−1^, or Δ*G* = −21.5 kJ·mol^−1^ at 37 °C.

Already during protein purification, apparent monomeric and dimeric species of Nck1-SH3.1 that do not interconvert at ambient temperature were observed and separated by gel-permeation chromatography (not shown, but see below). Only fractions of the monomer were used for initial biophysical studies, including crystallization. When a 1.14 mm sample of the Nck1-SH3.1 dimer in near-physiological buffer was chromatographed over a size-exclusion column after gentle overnight heating to 37 °C, separate Gaussian-shaped signals for dimeric and monomeric species were obtained (Fig. 9). These results indicate the presence of two defined species and facile separation of the swapped Nck1-SH3.1 dimer into monomers under mild conditions.

### Heat-induced domain swaps generate stable Nck1-SH3.1 monomers

**Figure 10.**
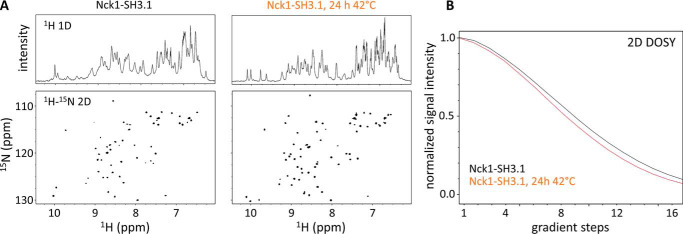
**NMR shows structured Nck1-SH3.1 species in solution.** *A*, the ^1^H (*top*) and ^15^N HSQC (*bottom*) spectra of the Nck1-SH3.1 dimer (*left panel*) show significant differences compared with spectra obtained from the identical sample after gentle heat treatment (24 h, 42 °C; *right panel*). Conformational differences in the two Nck1-SH3.1 species are reflected by numerous chemical shift perturbations of backbone (NH) resonance signals. Note the decreased line widths after heat treatment, indicating an increased rotational correlation time due to formation of monomers. The overall signal dispersion is not affected; thus, both species adopt stable structures, and no aggregation is present. *B*, DOSY detects differences in diffusion rates consistent with a roughly 2-fold mass difference. The faster signal decay for the heat-treated Nck1-SH3.1 indicates increased average diffusion rates for the molecules in this sample.

To test for the presence of unfolded SH3 domain, the conversion of Nck1-SH3.1 dimer to monomer by heat treatment was followed by NMR. Two-dimensional ^1^H-^15^N HSQC spectra were recorded for Nck1-SH3.1 before and after heat incubation (Fig. 10*A*). The spectral fingerprints of monomeric and dimeric Nck1-SH3.1 are quite different. Apart from very few residues at the termini, chemical shifts are significantly different for the two forms of Nck1-SH3.1. Both species have similar spectral dispersions, suggesting compact structures, in line with the crystal structures and excluding unfolded states of Nck1-SH3.1. A temperature-induced change of oligomeric state is also suggested by a concomitant decrease in signal line widths (*right-hand panel* of Fig. 10*A*), which indicate increased rotational correlation time due to increased molecular mass. Further support for dimerization is contributed by diffusion-ordered spectroscopy (DOSY), which detects an increase in diffusion rate after heating a dimeric Nck1-SH3.1 preparation to 42 °C for 24 h (Fig. 10*B*). Until this point, strong evidence for the monomeric and dimeric species has been gathered, but definite proof requires a direct method, which was applied next to study the different Nck1-SH3.1 species.

### AUC sedimentation of Nck1-SH3.1 identifies stable monomeric and dimeric species

**Figure 11.**
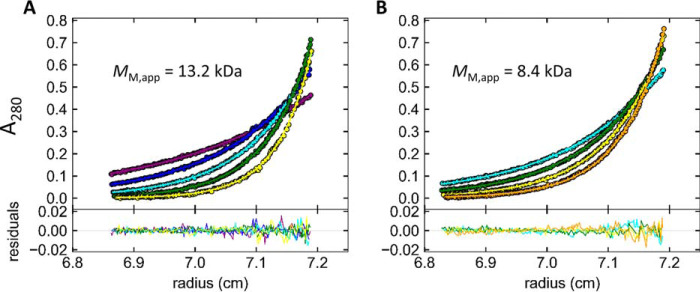
**Analytical ultracentrifugation of the Nck1-SH3.1 domain reveals dimers (*A*) and monomers (*B*) in solution.** Multispeed sedimentation equilibrium, radial, and time-invariant noise-subtracted absorption data at 280 nm are shown at the *top* as *dots* with global fits as *solid lines. Purple*, *blue*, *cyan*, *green*, *yellow*, and *orange* correspond to 20,000, 25,000, 30,000, 35,000, 40,000, and 45,000 rpm, respectively. The residuals of the fits are shown at the *bottom*. The sample in *B* was generated by preincubation of a 50 μm dimeric Nck1-SH3.1 preparation at 42 °C for 24 h to induce monomer formation. Use of 10 μm sample concentrations and detection at 250 nm did not significantly change the results, as expected (Fig. S3). For completeness and comparison with other detection wavelengths and sample concentrations, these panels are also present in Fig. S3.

Nck1-SH3.1 was analyzed by AUC in sedimentation equilibrium mode. Samples derived from the Nck1-SH3.1 dimer from gel permeation chromatography were indeed shown to be dimeric (Fig. 11 and Fig. S3). The measured values of molecular mass *M*_M_ = 13.1 kDa (68% confidence interval (CI) 11.4–15.0 kDa) and *M*_M_ = 13.2 kDa (68% CI 11.1–15.7 kDa) at 10 and 50 μm sample concentration, respectively, are within 5% of *M*_M_ expected for the Nck1-SH3.1 dimer (13.7 kDa). The high data quality is apparent from the low observed RMSD of <0.004 signal units in global fits to multispeed equilibrium data recorded at 280 and 250 nm. Together with the absence of any systematic deviation in the fits to the data, the analysis of the data warranted the assignment of a single, dimeric species. A sample pretreated at 42 °C for 24 h yielded a mass average of 8.4 kDa (68% CI 7.8–9.0 kDa), which is within 22% of the theoretical *M*_M_ of 6.9 kDa calculated from the amino acid sequence of Nck1-SH3.1 residues 4–59. The slight deviation from the expected value for the monomer *M*_M_ to larger values is presumably due to residual dimer after heating, in accordance with the gel permeation chromatography (Fig. 9). Accordingly, fitting the data with two discrete species of monomer and dimer, *M*_M_ for the monomer in the Sedphat Species Analysis model converged at *M*_M_ = 6.7 kDa for the monomer (*M*_M_ for the dimer was fixed at 13.2 kDa). Using a model for the analysis of monomer-dimer self-association in Sedphat and the molar extinction coefficients of Nck1-SH3.1 at 250/280 nm as input parameters, the value for the total concentration of Nck1-SH3.1 converged to 44.5 μm, which is reasonably close to the expected value of 50 μm. The equilibrium association constant was determined to be *K_a_* = 3377 m^−1^ (68% CI 2040–5310 m^−1^), in line with a similar value of 4151 m^−1^ from gel permeation (Fig. 9). The corresponding equilibrium dissociation constant from AUC is *K_d_* = 300 μm (68% CI 200–500 μm), which, despite very different methods, compares favorably with *K_d_* = 180 μm from gel permeation. From the fitted total concentration of 44.5 μm and the small value of *K_a_*, a molar ratio of [*M*]/[*D*] = 0.81 is calculated, which translates to 36 μm monomer and residual 4.2 μm dimer in the temperature-treated sample. Taken together, the sedimentation equilibrium data for two different sample concentrations over the entire range of increasing rotor velocities and the full time range of the experiment (∼150 h) could be fitted with single species that yielded the expected *M*_M_ of monomeric and dimeric Nck1-SH3.1. Thus, monomeric and domain-swapped Nck1-SH3.1 are distinct, stable species that do not interconvert over several days at ambient temperature but do so when warmed to 37–42 °C.

## Discussion

Nck has been found essential for T cell proliferation in response to weak antigens *i.e.* antigens with low affinity for the TCR, such as auto-antigens, but not in response to strong, potentially pathogen-derived antigens (7, 8). At the outset of this study, further development of AX-024, published as a PPI of the Nck1/CD3ϵ interaction (10), was anticipated. Unfortunately, we could not replicate all biological effects attributed to this compound. Our data show that AX-024 does not inhibit the interaction of Nck-SH3.1 and CD3ϵ. Whereas there is an inhibitory effect of AX-024 on T cell proliferation, we did not observe an effect on Zap70 phosphorylation, even though the assay was performed under comparable experimental conditions with primary human T cells as published previously (10). It is unclear at this stage whether the effect is donor-dependent, but in contrast to the results obtained in this study, it was published that AX-024 treatment reduces ZAP70 phosphorylation in anti-CD3–stimulated T cells to levels comparable with unstimulated T cells. Hence, the molecular mode of AX-024 action on T cells is unknown at present. A screen across a selectivity panel of 50 common “off-targets” revealed several alternative binding partners for AX-024. Although none of the corresponding genes is expressed in T cells, the result points to a propensity of AX-024 to bind to other proteins. Additional interaction partners and, thus, modes of action for AX-024 in T cells are therefore likely. We also could not reproduce the proliferation-inhibitory effect of CD3ϵ-derived peptides in cell cultures. Even though these cell-penetrating peptides bind to Nck1-SH3.1 *in vitro*, they did not inhibit T cell proliferation at concentrations below cytotoxic levels.

Unexpectedly, we did not observe an effect of the CD3ϵ APVA variant in Jurkat cells on TCR signaling, despite the fact that these changes abrogate, or at least severely decrease, the interaction between CD3ϵ and Nck1-SH3.1 *in vitro*. The results stand in contrast to previous findings in knock-in mice (11). Whereas the reasons for the discrepancy are currently unclear, they might be Jurkat cell–specific. It was shown that decreased amounts of Nck1 protein in Jurkat T cells resulted in an impairment of TCR-CD3–mediated activation involving a defective ERK phosphorylation pathway (19). It is conceivable that interaction of Nck and CD3ϵ via additional domains might be relevant for the modulation of TCR signaling *in vivo*.

As the concept of a PPI remained attractive, we focused our attention on delineating the PRS-binding site of Nck1-SH3.1. The affinity of CD3ϵ-derived peptides for Nck1-SH3.1 is characterized by *K_d_* values in the micromolar range. It has been shown that Nck binds to CD3ϵ of the TCR in a cooperative manner by employing its SH3.1 and SH2 domains (16). This cooperativity could substantially increase the overall affinity of Nck1 for CD3ϵ *in vivo*. Moreover, interactions of SH3 domains with residues outside the PRS are known to increase the affinity to their targets (20). The ITAM in CD3ϵ overlaps with the PRS that binds to Nck1-SH3.1. Tyr-188 is part of the PRS and must not be phosphorylated (21), whereas nearby Tyr-199 binds to the Nck1-SH2 domain in its phosphorylated form (16). The requirement of a nonphosphorylated Tyr-188 is explained by the crystal structure, as a phospho-Tyr-188 would disrupt the hydrogen bond to Glu-20 and the phosphoryl group would not fit into the PRS-binding site of Nck1-SH3.1. A similar conclusion was reached for the solution structure of Nck2-SH3.1 covalently connected to a cognate CD3ϵ sequence (15), although the specific interaction between Nck1 Glu-20 and CD3ϵ Tyr-188 was not mentioned in that study.

Because the SH3.1 and SH2 domains are located on opposite ends of Nck, the adapter molecule needs to collapse and form a circle to bind simultaneously to the closely spaced tyrosine residues in CD3ϵ PRS and ITAM. Such a collapse seems possible, given the sequences linking SH3.1/SH3.2 and SH3.3/SH2, which are predicted as largely disordered. The sequence ^260^EPSPPQCDY^268^ in the linker between SH3.3 and SH2 bears similarity to the PRS ^180^PPPVPNPDY^188^ specific to SH3.1. The sequence can be modeled into the SH3.1-binding site without steric hindrances. Although no experimental data are available to support this notion, a preorganized cyclic state of Nck1 where this sequence acts as a placeholder in the SH3.1 domain is conceivable.

Of the nine independent SH3.1 crystal structures obtained, seven were dimeric. Monomers and dimers of SH3.1 are stable in solution and can be isolated in pure form. However, interconversion is readily achieved by gentle heating, indicating similar stabilities of monomer and dimer. Solution NMR and AUC studies confirmed the results from crystal structures and gel permeation chromatography, raising the question of a likely biological function of a dimeric Nck.

Surveys of domain-swapped protein structures indicate that swaps of structural elements between monomers to form dimers, cyclic oligomers, or linear oligomers is common among proteins, with more than 100 proteins known to exhibit this feature (17, 18). To qualify as a domain swap, the monomer and oligomer need to be separable species. The “domain” can be a structural unit (*e.g.* an immunoglobulin domain in diabodies (22) and triabodies (23)). More often, however, secondary structure elements such as N- or C-terminal α-helices or β-strands are swapped (24–28). Some proteins are able to exchange larger parts, approaching half of their molecular masses (28, 29). Even double domain swaps occur (*e.g.* in catechol-*O*-methyltransferase (28) or in the streptococcal protein GB1 (30)), but these are much rarer. Common to all swapped oligomers is an additional protein-protein interface that is absent in the monomer. Depending on the energetic contribution of this interface, one of the species may be more stable than the other. This interface is small in the SH3.1 domain, in line with the similar stabilities of monomer and dimer.

**Figure 12.**
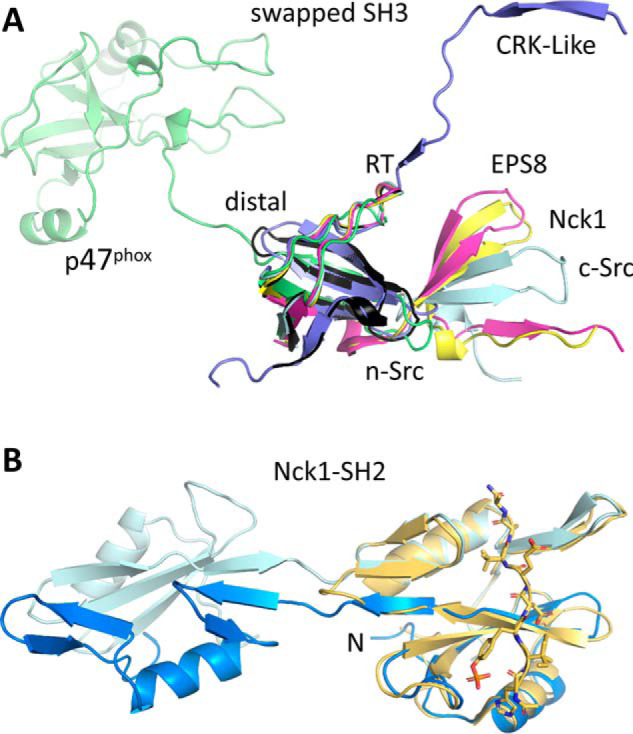
**Domains swaps in SH domains.** *A*, SH3 domains can swap from the RT, n-Src, and distal loops. Nck1-SH3.1 (*black* for the monomer, *yellow* for a swapped example) swaps from the n-Src loop, as do EPS8 (PDB entry 1I07; *magenta*) and c-Src (PDB entry 4JZ3; *pale cyan*). The SH3C domain of CRK-like (PDB entry 2BZY; *blue*) swaps from the RT loop, and the p47^phox^ SH3 domain (PDB entry 1UEC; *green*) swaps from the distal loop. *B*, the SH2 domain of Nck1 can also swap (PDB entry 2CI8; *hues* of *blue*), leading to the possibility of a double domain swap of this adapter protein. The phosphopeptide-bound monomer (PDB entry 2CIA; *yellow*) is shown for reference. The N terminus is *marked*.

Domain swaps in SH3 domains have been observed repeatedly (reviewed in Ref. 31) (Fig. 12*A*). The architectures of the resulting dimers depend on the site of the swap. All of the three hallmark loops in SH3 domains—the RT, n-Src, and distal loops—can serve as hinge regions for a swap. The RT loop is the site of domain swap in an extended SH3 domain dimer of human adaptor protein CRK-like (32). In the SH3_A_ domain of human p47^phox^, the swap occurs via the distal loop (33, 34) (Fig. 12*A*). Although truncation may favor domain swapping (17), the example of p47^phox^ shows that swapping is not a propensity limited to isolated SH3 domains, as an extra domain of 180 residues of this regulatory subunit of the phagocyte NADPH oxidase is also present in the crystal structure (33). Most of the observed SH3 domain swaps, however, occur in the n-Src loop. Examples other than Nck1-SH3.1 include epidermal growth factor receptor kinase substrate 8 (EPS8) (35) and c-Src (36, 37), which accordingly form dimers of similar topologies to Nck1-SH3.1 (Fig. 12*A*). The SH3 domain of chicken c-Src not only associates into dimers, but also can form amyloids at mildly acidic pH, indicating substantial structural plasticity of this small domain. That the SH3 fold is inherently flexible is underscored by the observation that high PEG concentrations can also induce the dimeric c-Src SH3 structure (37). Small-angle X-ray scattering has detected concentration- and pH-dependent dimerization of Nck2-SH3.1 (38). Molecular dynamics simulations support a general propensity of SH3 domains to form dimers and amyloids (39), and a single point variant in the RT loop of the c-Src SH3 domain was shown to form fibrils at neutral pH (31). Domain swapping has been implicated in the formation of disease-causing fibrillary aggregates, indicating that swaps do occur *in vivo* and that there is evolutionary pressure on this propensity (reviewed in Refs. 40 and 31 for SH3 domains).

Not only SH3 domains, but also SH2 domains present in adapter and signaling proteins can swap, which is of particular relevance for Nck, as it contains both types of domains. The C-terminal SH2 domain of Nck has been observed to swap (41) (Fig. 12*B*). Although solution methods did not detect dimeric Nck1-SH2 species (41), this nonetheless raises the interesting possibility of a double domain swap leading to higher-order oligomers of Nck. Self-assembly of Nck, mediated by the linker region between SH3.1 and SH3.2, was reported previously as a possible mechanism for phase separation of Nck complexes with N-WASP and p-nephrin (42). Other examples of swapped SH2 domains include p59-Fyn kinase, where the swap abolishes, for structural reasons, phosphopeptide binding (43). By contrast, a swapped dimer of the SH2 domain from the adaptor protein Grb2 (44, 45) retains phosphopeptide affinity, albeit with 4–13-fold reduced *K_d_* values (44).

Apart from pathogenic fibril formation, the physiological relevance of domain swaps is often questionable. Some domain-swapped structures have been assigned as likely artifacts due to protein truncation (33) or extremes of pH (28) and ionic strength (41). However, there is increasing evidence of biologically relevant domain swaps, some of which are ligand-dependent. For example, GSH regulates the monomer-dimer equilibrium of glyoxalase I (46). Similarly, a phosphopeptide influences the monomer-dimer equilibrium of p13*^suc1^*, a cell-cycle regulator in fission yeast (47). In general, domain swaps may serve for functional interconversion between monomers and dimers, and swapping has been implicated as a possible mechanism for oligomer evolution (48). Polymerization by domain swapping is a mechanism for self-assembly of the bacterial flagellar motor (49). Whereas the presence of stable Nck1-SH3.1 monomers and dimers in solution at ambient temperature and physiological ionic strength would argue in favor of Nck1 swaps *in vivo*, whether a swapped dimeric or even oligomeric form of Nck1 is of biological significance remains to be determined.

## Experimental procedures

### Generation of a mutant Jurkat cell line with abrogated CD3ϵ/Nck1-SH3.1 interaction

Jurkat cells bearing the PPVP to APVA double mutation in the PRS of CD3ϵ were generated using the CRISPR-Cas9 system. The guide RNA target sequence CCACCTGTTCCCAACCCAGACTA (protospacer-adjacent motif (PAM) underlined) was selected using the CRISPOR web tool (50) and cloned in CRISPR Nuclease Vector according to vendor instructions (Thermo Fisher Scientific, catalog no. A21178). Plasmid and single-stranded oligo-DNA (ssODN) repair template were electroporated together into Jurkat cells using the Neon system (single pulse for 20 ms of 1600 V; Thermo Fisher Scientific). The ssODN repair template atgtttcccctccttcctccgcagGACAAAACAAGGAGAGGCCA**G**CACCTGTT**G**CCAACCCAGACTATGAGgtaacgtgggatag contained two phosphorothioate linkages between the first and last two nucleotides (Microsynth AG, Switzerland). In addition, two nucleotide changes (in boldface type) that modify the two proline residues in the P*XX*P motif of the SH3.1-binding sequence in Nck1 to alanine were included. One of the two mutations (CCA → **G**CA) also altered the PAM and at the same time created a new CaiI/AlwNI restriction site (CAGNNNCTG) that was used to screen for heterozygous and homozygous clones. Two days after electroporation, single cells were sorted in three 96-well plates by FACS based on the presence of orange fluorescent protein. An aliquot of the cells was also sorted in a tube. This heterogeneous pool of sorted cells served to verify both the Cas9-mediated cleavage using T7E1 endonuclease (New England Biolabs, catalog no. M0302L) and the homology directed repair by CaiI restriction (Thermo Fisher Scientific, catalog no. FD1394). For both assays, genomic DNA was extracted from the cells, and the sequence targeted within the CD3ϵ gene was amplified by PCR using forward and reverse primers 5′-TTGCCATTCTCTATCTGGGTC-3′ and 5′-GGGTTGTAATGGAAGCCCTGA-3′, respectively. About 2 weeks after single cell sorting, growing clones were screened for the mutations introduced by DNA/extraction and restriction. Five different homozygous clones were identified and verified by DNA sequencing. Only clones expressing *CD3* levels similar to nonmanipulated Jurkat cells were used.

### Stimulation of Jurkat clones and staining for phosphorylated ERK and Zap70

Jurkat and APVA mutant Jurkat cells were starved overnight and added to 96-well plates coated with anti-CD3 (clone OKT3, BioLegend, lot B235453) on ice. Cells treated with PMA/ionomycin (cell activation mixture, BioLegend) and H_2_O_2_ (0.03% final concentration, Merck) were used as controls. Cells were stimulated for various times before fixation (BD Cytofix fixation buffer). Upon permeabilization (TruePhos buffer, BioLegend), the cells were stained with phosphorylation-sensitive antibodies P-ERK-BV421 (clone 6B8B69, BioLegend) and P-Zap70-PE (clone 65E4, CST). Flow cytometry was performed on a BD Fortessa cell analyzer, and the data were evaluated using FlowJo software.

### Primary T cell proliferation

Fresh human peripheral blood mononuclear cells (PBMCs) from healthy donors (*n* = 4) were prepared from buffy coats. *CD3*^+^ T cells were isolated by immunomagnetic separation (negative selection, StemCell, lot 18A87710), carboxyfluorescein succinimidyl ester (CFSE)-labeled, resuspended in StemCell Immunocult^TM^-XF T cell expansion medium, and treated with compounds or vehicle for 2 h at room temperature. The isolated CD3^+^ T cells were stained for CD3, CD4, and CD8, and their purity was assessed by flow cytometry. Cells were then cultured in a 96-well flat-bottomed plate (10^5^ cells/well), precoated for 1 h at 37 °C with 0.2 μg·ml^−1^ plate-bound anti-*CD3* (clone OKT3, BioLegend). Precoated plates were also blocked with a 5% solution of BSA for 1 h at 37 °C prior to the addition of cells to wells. Cells were cultured in the presence of AX-024, rapamycin, peptide 11Rwt, peptide 11R85, peptide 11Rscr (derived from Ref. 11; see below for sequences), or vehicle for 4 days. At the end of culture, cells were stained for viability, and CFSE dilution was assessed by flow cytometry (with gating on CD4 *versus* CD8).

### Stimulation and Zap70 phosphorylation of primary T cells

Human PBMCs from healthy donors were prepared from 50 ml of fresh blood with Ficoll in Leucosep tubes. The tubes were centrifuged at room temperature (30 min, 800 × g) without brake. Isolated cells were washed and staved overnight. The cells were treated with AX-024 or vehicle and incubated for 1 h at 37 °C followed by further incubation on ice. 10, 25, or 50 μg·ml^−1^ anti-CD3-biotin (clone OKT3, BioLegend) was added to the cells on ice for 15 min before neutravidin was added at a final concentration of 20 μg·ml^−1^ for cross-linking. After stimulation for 3 min at 37 °C, cells were fixed (BD Cytofix fixation buffer) and permeabilized (TruePhos buffer, BioLegend). Staining was performed with CD3-PerCP (clone SK7, BD), CD4-BUV395 (clone SK3, BD), CD8-BV605 (clone RPAT8, BioLegend), and Zap70-PE (clone 65E4, CST). Flow cytometry and data analysis were done as described above.

### CD3ϵ-derived peptides and AX-024

C-terminally amidated peptides RGQNKERPPPVPNPDY-NH_2_ and variant RGQNKERP**A**PV**A**NPDY-NH_2_ (11) for SPR studies were obtained from Bachem (Bubendorf, Switzerland), and all others were from Biosynthan (Berlin, Germany). These included peptides 11Rwt of sequence R_11_-G_3_-RGYNKERPPPVPNPDY, 11R085 of sequence R_11_-G_3_-Q(dK)KECPPPVPKRDY, and 11Rscr of sequence R_11_-G_3_-PKVRECPDYK(dK)PQP, where (dK) is the *R*-enantiomer of K. The R_11_ polyarginine tag connected by a triglycine linker to the CD3-relevant sequences was used before to enable cell penetration of the peptides (11). AX-024, or 1-((4-(4-fluorophenyl)-6-methoxy-2H-chromen-3-yl)methyl)pyrrolidine hydrochloride, was purchased from Axon MedChem BV (Groningen, Netherlands) and submitted for in-house quality control by NMR and MS to verify its published chemical structure.

### Protein constructs, production, and purification of the Nck1-SH3.1 domain

*Escherichia coli*-optimized synthetic genes coding for human Nck1-SH3.1 residue ranges 5–59 and 1–61 followed by a stop codon were ordered from GeneScript and NcoI/NotI cloned into a modified pET28a(+) vector (Novagen). The resulting constructs code for an N-terminal His_10_ tag fused to an AviTag for biotinylation, followed by a tobacco etch virus (TEV) cleavage site. Alternatively, a SUMO fusion was included between the His and Avi tags. Production of the Nck1-SH3.1 domain (residue ranges 4–59 and 1–61) was thus tested as His_10_-Avi-TEV and as His_6_-SUMO-Avi-TEV fusion proteins. 3-ml cultures of transformed *E. coli* BL21 (DE3) cells grown in lysogeny broth or TB medium were tested for protein production at 37 and 20 °C after induction with 0.5 mm IPTG. Based on protein mass and solubility, the constructs with a SUMO tag proved suitable for large-scale protein production and purification. 100 ml of TB medium, supplemented with 75 μg·ml^−1^ kanamycin for vector maintenance, was inoculated with 20 ml of an overnight culture and grown at 37 °C to an OD_600_ of 0.35. The culture was cooled to 20 °C, and protein production was induced with 0.5 mm IPTG at OD_600_ = 0.6–0.8. After 16–18 h, cells were harvested by centrifugation and stored at −80 °C.

Both His_6_-SUMO-Avi-TEV-Nck1(1–61) and His_6_-SUMO-Avi-TEV-Nck1(4–59) were purified following the same protocol. About 25 g of *E. coli* cells were resuspended in 100 ml of buffer A (50 mm HEPES/NaOH, pH 7.8, 500 mm NaCl, 1 mm tris-(2-carboxyethyl)phosphine HCl) (TCEP), supplemented with 10 mm imidazole, 1 mm MgCl_2_, DNase I (Roche Applied Science, catalog no. 04716728001), and EDTA-free protease inhibitor mixture (Roche Applied Science, catalog no. 05056489001; 1 tablet/50 ml). Cells were lysed at 80 megapascals pressure in a Microfluidizer LM20 (Microfluidics), and insoluble material was removed by centrifugation (Beckman Coulter Avanti JXN-20, JA-25.50, 40,000 × *g*, 4 °C). The supernatant was passed through a 0.22-μm filter and loaded onto a Ni^2+^-Sepharose column (HisTrap HP 5 ml, GE Healthcare, catalog no. 17-5247-01), equilibrated with buffer A plus 10 mm imidazole. The column was washed with this buffer, and bound His_6_-tagged protein was eluted by applying a linear gradient of 0–250 mm imidazole in buffer A over 100 ml. Fractions containing His_6_-SUMO-Avi-TEV-Nck1 fusion protein were pooled and dialyzed against buffer A at 4 °C (Spectra/Por Dialysis Membrane; molecular weight cutoff 3500). For experiments that required cleaved fusion proteins, SUMO-protease (Thermo Fisher Scientific, catalog no. 12588018) or TEV protease were included in the dialyses. His-tagged proteins were removed from cleaved Nck1 fragments by chromatography on a 5-ml Ni^2+^-Sepharose column, equilibrated with buffer A. The flow-through was concentrated using Amicon Ultra-15 centrifugal filters with an Ultracel-3K membrane (Millipore, catalog no. UFC900324) at 4500 × *g* and then loaded onto a size-exclusion column (S75 10/300 GL, GE Healthcare, catalog no. 17-5174-01) equilibrated with 25 mm HEPES/NaOH, pH 7.8, 150 mm NaCl. Fractions containing apparent monomeric and dimeric Nck1 fragments were pooled separately. For brevity, we refer to just “monomer” and “dimer” throughout, even though the identity of the monomers and dimers as stable, separate species was confirmed only later by analytical ultracentrifugation (AUC).

Avi-TEV-tagged Nck1 obtained from treatment of fusion proteins with SUMO-protease was biotinylated *in vitro* using the BirA reaction kit (Avidity, catalog no. BirA500). Reaction conditions were chosen as recommended by the manufacturer. To remove surplus reaction components, the reaction mixture was desalted on a 5-ml HiTrap desalting column (GE Healthcare, catalog no. 29-0486-84) in 50 mm HEPES/NaOH, pH 7.8, 150 mm NaCl, 1 mm TCEP, 10% (v/v) glycerol.

### SPR

SPR experiments were conducted on a T200 Biacore instrument (GE Healthcare, Uppsala, Sweden) at 20 °C in 10 mm HEPES/NaOH, pH 7.8, 150 mm NaCl, 1 mm TCEP. His_10_-Avi-TEV-Nck1-SH3.1 (residues 1–61, *M*_M_ 11,847 Da) was biotinylated and immobilized via streptavidin on the surface of either a CAP or a SA sensor, prepared according to the manufacturer's instructions (GE Healthcare). Streptavidin is immobilized on the CAP sensor by way of double-strand DNA formation. A ssDNA is covalently coupled to the sensor surface, and a complementary DNA strand, covalently linked to streptavidin, is hybridized. To regenerate the surface, the DNA duplex is dissociated by washing with high-ionic strength solution. Thus, after each titration, fresh biotinylated Nck1-SH3.1 was immobilized on the sensor surface. For CD3ϵ peptide-binding experiments, the surface density of the Nck1-SH3.1 domain was ∼250 response units (RU), whereas for binding experiments with AX-024 and APVA variant peptides, the surface density of Nck1-SH3.1 was increased to 1260 RU. After Nck1-SH3.1 immobilization, remaining free streptavidin on the CAP sensor was blocked using a 500 nm biotin solution. The reference channel for binding studies contained the same streptavidin-coated surface blocked with biotin.

Binding of the CD3ϵ-derived peptide RGQNKERPPPVPNPDY-NH_2_ (CD3ϵ residues 173–188; *M*_M_ 1864.1 Da) or of the APVA variant RGQNKERPAPVANPDY-NH_2_ (*M*_M_ 1811.1 Da) to the immobilized Nck1-SH3.1 domain was monitored in a concentration-dependent manner between 0.4 and 100 μm CD3ϵ peptide (200 μm in the case of the APVA variant peptide). The immobilized Nck1-SH3.1 domain was contacted with different peptide concentrations over an association phase of 60 s, followed by a dissociation phase of 120 s. Five replicates were performed for each CD3ϵ concentration (Table S1), whereas for the qualitative assessment of the APVA variant, only a single titration was performed. Binding of the small molecule AX-024 to the Nck1-SH3.1 domain was tested both at a single concentration of 100 μm and in competition with CD3ϵ peptide (100 μm each).

SPR equilibrium signals were corrected for the signal in the reference channel and buffer contributions. The resulting SPR amplitudes were plotted as a function of peptide concentration, and a simple binding equation describing a Langmuir adsorption isotherm was fit to the data (GraphPad Prism) to extract the equilibrium dissociation constant *K_d_*.

(Eq. 1) $$RU=RU_{\max}\cdot \frac{\left[\text{peptide}\right]}{\left[\text{peptide}\right]+K_{d}}$$

### NMR measurements

NMR spectra were recorded on a Bruker 600-MHz Avance NEO spectrometer equipped with a cryogenic QCI probe head at 27 °C in 25 mm HEPES/NaOH, pH 7.4, 150 mm NaCl. The Topspin 4.0 software (Bruker, Fällanden) was used for spectrometer operation and data processing. For ^1^H ligand-observed measurements, a relaxation filtered pulse sequence utilizing a spinlock of 50 ms was applied. 1D spectra were acquired with 128 scans, a sweep width of 26 ppm, and an acquisition time of 2 s. 2D ^1^H-^15^N HSQC spectra with natural ^15^N isotope abundance were recorded over 72 h with a spectral width of 16 ppm and 35 ppm in the proton and nitrogen dimensions, respectively, with 256 increments (complex) and 1000 scans per increment. A relaxation delay of 1 s was used between scans. For the DOSY measurements (reviewed in Ref. 51), the Bruker pulse sequence ledbpgp2s with a diffusion delay of 50 ms and a gradient length of 600 μs was used. The gradient strength was varied from 5 to 95% to record 16 increments of 512 scans with a sweep width of 18 ppm and a relaxation delay of 1 s.

Samples for ligand-observed measurements were prepared in 25 mm sodium phosphate buffer, pH 6.5, 150 mm NaCl. One sample contained 50 μm AX-024, and the other contained 50 μm CD3ϵ peptide. After acquisition of their 1D ^1^H NMR spectra, 10 μm Nck1-SH3.1 was added, and the spectra were retaken. These experiments were performed with dimeric as well as heat-induced monomeric Nck1-SH3.1. Protein-observed 2D ^1^H-^15^N HSQC spectra were collected from 750 μm Nck1-SH3.1 solutions. Spectra were recorded for dimeric and monomeric apo Nck1-SH3.1, as well as for monomeric Nck1-SH3.1 in the presence of 1 mm CD3ϵ peptide.

### Crystallization and structure determination

The sitting drop setup at 22 °C was used for all crystallization trials. Droplets contained 30–70% (v/v) 5–20 mg·ml^−1^ Nck1-SH3.1 in a total drop volume of 400 nl that was equilibrated against 40 μl of reservoir. A summary of crystallization conditions for the Nck1-SH3.1 domain is provided in Table S3. Crystals of Nck1-SH3.1 in complex with the CD3ϵ-derived peptide Ac-PPPVPNPDY-NH_2_ (2-fold molar excess; *M*_M_ = 1109.3 Da; >95% purity as judged by HPLC) grew from 0.1 m BisTris/HCl, pH 6.5, 2 m (NH_4_)_2_SO_4_. Crystals of monomeric and dimeric SH3.1 grew over a pH range of 5–8 and a variety of precipitants. Depending on the crystallization condition, crystals were either flash-cooled directly in liquid nitrogen or cryoprotected with paraffin oil prior to vitrification. Diffraction data were collected at 100 K at beam line X10SA (PXII) of the Swiss Light Source using a Pilatus II pixel detector (Dectris, Villigen). Parameters were 1-Å wavelength, 0.25-s exposure time, and 0.2° oscillation width (200° total range). Images were processed with XDS (52), scaled with AIMLESS, and treated for anisotropy using ellipsoidal masking in STARANISO (RRID:SCR_018362). The high-resolution cut-off was determined as CC_½_ > 0.3 in the high-resolution shell (53, 54), which is the currently recommended procedure. Anisotropic distribution of reflections leads to low completeness, but with valid data, in the high-resolution shells for some data sets, which is why the 100% criterion (55) for the high-resolution limit was included as a comparison (Table S4). It is the resolution of a 100% complete isotropic data set with the same number of reflections as those measured. Whereas none of the crystals diffracted to worse than 2 Å resolution, some approached near-atomic resolution (Table S4). Data sets were phased by molecular replacement with PHASER (56). The search model for the first data set was the human hematopoietic cell kinase SH3 domain (PDB entry 1QCF). Later data sets were phased using in-house Nck1-SH3.1 coordinates. Models were built into electron density using COOT (57) and refined with REFMAC5 (58), except for 5QU6, which was refined with PHENIX (59). Anisotropic *B*-values were refined for resolutions better than 1.3 Å. For all others, TLS domain definitions were determined using PHENIX, and TLS parameters were refined in addition to individual *B*-values. No NCS restraints were needed for stable refinements. The CCP4 program suite (55) was used for all other crystallographic calculations. Table S4 summarizes data collection and refinement statistics. Structure figures were created with PyMOL (Schrödinger).

### AUC

All experiments were conducted at 20 °C using a Proteome Lab XLI analytical ultracentrifuge equipped with an An-60Ti rotor (Beckman Coulter). Nck1-SH3.1 (residues 4–59, dimer peak from SEC) was dialyzed against 25 mm HEPES/NaOH, pH 7.4, 150 mm NaCl (ρ = 1.0622 g·ml^−1^ at 20 °C) and filled in SedVel60K charcoal-filled Epon centerpieces (Spin Analytical; 1.2- or 0.3-cm optical path length for 10 or 50 μm, respectively). The sample volume was 120 μl for 1.2-cm and 30 μl for 0.3-cm centerpiece diameter. One sample was incubated at 50 μm and 42 °C for 24 h to induce monomer formation starting from the dimer. Prior to initiating AUC runs, the rotor with the loaded sample cells was kept at 20 °C in the centrifuge for 2 h for thermal equilibration of the experimental setup. Samples were measured in multispeed sedimentation equilibrium mode at 20,000, 25,000, 30,000, 35,000, 40,000, and 45,000 rpm, with equilibration times of 30 h for the first and 24 h for subsequent rotor speeds. Radial scans were measured by absorbance at 250 and 280 nm (10-μm radial step size, 25 repeats/scan). The partial specific volume of 0.733 ml·g^−1^ was calculated from the amino acid sequence of Nck1-SH3.1. Sedimentation equilibrium data were analyzed with Sedphat (60). The molecular mass was determined with the species analysis module in Sedphat. Sedimentation equilibrium profiles were plotted with GUSSI (61). Small root mean square deviation of global fits of the multispeed equilibrium data and absence of systematic deviations in the residuals allowed assignment of single dimeric or monomeric species for the individual samples. The 68.3% CI of the best-fitting values for the molecular mass were calculated by the error surface projection mode on global fits of data recorded at 250 and 280 nm, as implemented in Sedphat. The monomer and dimer concentrations were estimated using the solution to the quadratic equation describing the 2 M ⇄ D equilibrium,

(Eq. 2) $$\left[M\right]=\frac{-1+\sqrt{1+8\cdot K_{a}\cdot \left[{\text{M}}_{0}\right]}}{4\cdot K_{a}}$$

where [*M*] is the monomer concentration at equilibrium, [M_0_] is the total protein concentration, and *K_a_* is the fitted equilibrium association constant. The dimer concentration was accordingly calculated as [*D*] = ½·([M_0_] − [*M*]).

## Data availability

The atomic coordinates and structure factors have been deposited in the Protein Data Bank under accession codes 5QU1–5QU8 and 5QUA. All other data are contained within the article.

## Supplementary Material

Supporting Information

## Footnotes

4 The abbreviations used are:

MHC: major histocompatibility complex
AUC: analytical ultracentrifugation
DOSY: diffusion ordered spectroscopy
Nck1: non-catalytic region of tyrosine kinase adaptor protein 1
Nck1-SH3.1: first SH3 domain in human Nck1
ssODN: single-stranded oligo-DNA
SH2 and SH3: Src homology 2 and 3, respectively
SPR: surface plasmon resonance
ZAP70: ζ chain of T cell receptor–associated protein kinase 70
TCR: T cell receptor
ITAM: immunoreceptor tyrosine-based activation motif
WASP: Wiskott–Aldrich syndrome protein
PPI: protein-protein inhibitor
1D and 2D: one- and two-dimensional, respectively
RMSD: root mean square deviation
CI: confidence interval
EPS8: epidermal growth factor receptor kinase substrate 8
PAM: protospacer-adjacent motif
ERK: extracellular signal–regulated kinase
PMA: phorbol 12-myristate 13-acetate
PBMC: peripheral blood mononuclear cell
CFSE: carboxyfluorescein succinimidyl ester
TEV: tobacco etch virus
IPTG: isopropyl 1-thio-β-d-galactopyranoside
TCEP: tris-(2-carboxyethyl)phosphine HCl
PDB: Protein Data Bank
P-: phosphorylated
mAU: milli-absorbance units
PRS: proline-rich sequence
HCK: hematopoietic cell kinase
BisTris: 2-[bis(2-hydroxyethyl)amino]-2-(hydroxymethyl)propane-1,3-diol.

## References

[1] Love P. E., and Hayes S. M. (2010) ITAM-mediated signaling by the T-cell antigen receptor. Cold Spring Harb. Perspect. Biol. 2, a002485 10.1101/cshperspect.a002485 20516133 PMC2869518

[2] Ngoenkam J., Schamel W. W., and Pongcharoen S. (2018) Selected signalling proteins recruited to the T-cell receptor-CD3 complex. Immunology 153, 42–50 10.1111/imm.12809 28771705 PMC5721247

[3] Lettau M., Pieper J., and Janssen O. (2009) Nck adapter proteins: functional versatility in T cells. Cell Commun. Signal. 7, 1 10.1186/1478-811X-7-1 19187548 PMC2661883

[4] Lettau M., Pieper J., Gerneth A., Lengl-Janssen B., Voss M., Linkermann A., Schmidt H., Gelhaus C., Leippe M., Kabelitz D., and Janssen O. (2010) The adapter protein Nck: role of individual SH3 and SH2 binding modules for protein interactions in T lymphocytes. Protein Sci. 19, 658–669 10.1002/pro.334 20082308 PMC2867007

[5] Ngoenkam J., Paensuwan P., Preechanukul K., Khamsri B., Yiemwattana I., Beck-García E., Minguet S., Schamel W. W., and Pongcharoen S. (2014) Non-overlapping functions of Nck1 and Nck2 adaptor proteins in T cell activation. Cell Commun. Signal. 12, 21 10.1186/1478-811X-12-21 24670066 PMC3977700

[6] Bladt F., Aippersbach E., Gelkop S., Strasser G. A., Nash P., Tafuri A., Gertler F. B., and Pawson T. (2003) The murine Nck SH2/SH3 adaptors are important for the development of mesoderm-derived embryonic structures and for regulating the cellular actin network. Mol. Cell. Biol. 23, 4586–4597 10.1128/MCB.23.13.4586-4597.2003 12808099 PMC164855

[7] Tailor P., Tsai S., Shameli A., Serra P., Wang J., Robbins S., Nagata M., Szymczak-Workman A. L., Vignali D. A., and Santamaria P. (2008) The proline-rich sequence of CD3ϵ as an amplifier of low-avidity TCR signaling. J. Immunol. 181, 243–255 10.4049/jimmunol.181.1.243 18566390 PMC2665931

[8] Roy E., Togbe D., Holdorf A. D., Trubetskoy D., Nabti S., Küblbeck G., Klevenz A., Kopp-Schneider A., Leithäuser F., Möller P., Bladt F., Hämmerling G., Arnold B., Pawson T., and Tafuri A. (2010) Nck adaptors are positive regulators of the size and sensitivity of the T-cell repertoire. Proc. Natl. Acad. Sci. U.S.A. 107, 15529–15534 10.1073/pnas.1009743107 20709959 PMC2932578

[9] Borroto A., Abia D., and Alarcón B. (2014) Crammed signaling motifs in the T-cell receptor. Immunol. Lett. 161, 113–117 10.1016/j.imlet.2014.05.007 24877875

[10] Borroto A., Reyes-Garau D., Jiménez M. A., Carrasco E., Moreno B., Martínez-Pasamar S., Cortés J. R., Perona A., Abia D., Blanco S., Fuentes M., Arellano I., Lobo J., Heidarieh H., Rueda J., et al (2016) First-in-class inhibitor of the T cell receptor for the treatment of autoimmune diseases. Sci. Transl. Med. 8, 370ra184 10.1126/scitranslmed.aaf2140 28003549

[11] Borroto A., Arellano I., Blanco R., Fuentes M., Orfao A., Dopfer E. P., Prouza M., Suchànek M., Schamel W. W., and Alarcón B. (2014) Relevance of Nck-CD3 ϵ interaction for T cell activation *in vivo*. J. Immunol. 192, 2042–2053 10.4049/jimmunol.1203414 24470497

[12] Noguchi H., Matsushita M., Okitsu T., Moriwaki A., Tomizawa K., Kang S., Li S. T., Kobayashi N., Matsumoto S., Tanaka K., Tanaka N., and Matsui H. (2004) A new cell-permeable peptide allows successful allogeneic islet transplantation in mice. Nat. Med. 10, 305–309 10.1038/nm994 14770176

[13] Santiveri C. M., Borroto A., Simon L., Rico M., Alarcón B., and Jiménez M. A. (2009) Interaction between the N-terminal SH3 domain of Nck-α and CD3-ϵ-derived peptides: non-canonical and canonical recognition motifs. Biochim. Biophys. Acta 1794, 110–117 10.1016/j.bbapap.2008.09.016 18955169

[14] Schindler T., Sicheri F., Pico A., Gazit A., Levitzki A., and Kuriyan J. (1999) Crystal structure of Hck in complex with a Src family-selective tyrosine kinase inhibitor. Mol. Cell 3, 639–648 10.1016/S1097-2765(00)80357-3 10360180

[15] Takeuchi K., Yang H., Ng E., Park S. Y., Sun Z. Y., Reinherz E. L., and Wagner G. (2008) Structural and functional evidence that Nck interaction with CD3ϵ regulates T-cell receptor activity. J. Mol. Biol. 380, 704–716 10.1016/j.jmb.2008.05.037 18555270 PMC2577852

[16] Paensuwan P., Hartl F. A., Yousefi O. S., Ngoenkam J., Wipa P., Beck-Garcia E., Dopfer E. P., Khamsri B., Sanguansermsri D., Minguet S., Schamel W. W., and Pongcharoen S. (2016) Nck binds to the T cell antigen receptor using its SH3.1 and SH2 domains in a cooperative manner, promoting TCR functioning. J. Immunol. 196, 448–458 10.4049/jimmunol.1500958 26590318

[17] Liu Y., and Eisenberg D. (2002) 3D domain swapping: as domains continue to swap. Protein Sci. 11, 1285–1299 10.1110/ps.0201402 12021428 PMC2373619

[18] Rousseau F., Schymkowitz J., and Itzhaki L. S. (2012) Implications of 3D domain swapping for protein folding, misfolding and function. Adv. Exp. Med. Biol. 747, 137–152 10.1007/978-1-4614-3229-6_9 22949116

[19] Yiemwattana I., Ngoenkam J., Paensuwan P., Kriangkrai R., Chuenjitkuntaworn B., and Pongcharoen S. (2012) Essential role of the adaptor protein Nck1 in Jurkat T cell activation and function. Clin. Exp. Immunol. 167, 99–107 10.1111/j.1365-2249.2011.04494.x 22132889 PMC3248091

[20] Teyra J., Huang H., Jain S., Guan X., Dong A., Liu Y., Tempel W., Min J., Tong Y., Kim P. M., Bader G. D., and Sidhu S. S. (2017) Comprehensive analysis of the human SH3 domain family reveals a wide variety of non-canonical specificities. Structure 25, 1598–1610.e3 10.1016/j.str.2017.07.017 28890361

[21] Kesti T., Ruppelt A., Wang J. H., Liss M., Wagner R., Taskén K., and Saksela K. (2007) Reciprocal regulation of SH3 and SH2 domain binding via tyrosine phosphorylation of a common site in CD3ϵ. J. Immunol. 179, 878–885 10.4049/jimmunol.179.2.878 17617578

[22] Perisic O., Webb P. A., Holliger P., Winter G., and Williams R. L. (1994) Crystal structure of a diabody, a bivalent antibody fragment. Structure 2, 1217–1226 10.1016/S0969-2126(94)00123-5 7704531

[23] Pei X. Y., Holliger P., Murzin A. G., and Williams R. L. (1997) The 2.0-Å resolution crystal structure of a trimeric antibody fragment with noncognate VH-VL domain pairs shows a rearrangement of VH CDR3. Proc. Natl. Acad. Sci. U.S.A. 94, 9637–9642 10.1073/pnas.94.18.9637 9275175 PMC23241

[24] Mazzarella L., Capasso S., Demasi D., Di Lorenzo G., Mattia C. A., and Zagari A. (1993) Bovine seminal ribonuclease: structure at 1.9 Å resolution. Acta Crystallogr. D Biol. Crystallogr. 49, 389–402 10.1107/S0907444993003403 15299514

[25] Green S. M., Gittis A. G., Meeker A. K., and Lattman E. E. (1995) One-step evolution of a dimer from a monomeric protein. Nat. Struct. Biol. 2, 746–751 10.1038/nsb0995-746 7552745

[26] Spinelli S., Desmyter A., Frenken L., Verrips T., Tegoni M., and Cambillau C. (2004) Domain swapping of a llama VHH domain builds a crystal-wide β-sheet structure. FEBS Lett. 564, 35–40 10.1016/S0014-5793(04)00304-7 15094039

[27] Baxter E. L., Jennings P. A., and Onuchic J. N. (2012) Strand swapping regulates the iron-sulfur cluster in the diabetes drug target mitoNEET. Proc. Natl. Acad. Sci. U.S.A. 109, 1955–1960 10.1073/pnas.1116369109 22308404 PMC3277509

[28] Ehler A., Benz J., Schlatter D., and Rudolph M. G. (2014) Mapping the conformational space accessible to catechol-*O*-methyltransferase. Acta Crystallogr. D Biol. Crystallogr. 70, 2163–2174 10.1107/S1399004714012917 25084335 PMC4118827

[29] Rufer A. C., Kusznir E., Burger D., Stihle M., Ruf A., and Rudolph M. G. (2018) Domain swap in the C-terminal ubiquitin-like domain of human doublecortin. Acta Crystallogr. D Struct. Biol. 74, 450–462 10.1107/S2059798318004813 29717716

[30] Kirsten Frank M., Dyda F., Dobrodumov A., and Gronenborn A. M. (2002) Core mutations switch monomeric protein GB1 into an intertwined tetramer. Nat. Struct. Biol. 9, 877–885 10.1038/nsb854 12379842

[31] Cámara-Artigas A. (2016) Crystallographic studies on protein misfolding: domain swapping and amyloid formation in the SH3 domain. Arch. Biochem. Biophys. 602, 116–126 10.1016/j.abb.2016.02.024 26924596

[32] Harkiolaki M., Gilbert R. J., Jones E. Y., and Feller S. M. (2006) The C-terminal SH3 domain of CRKL as a dynamic dimerization module transiently exposing a nuclear export signal. Structure 14, 1741–1753 10.1016/j.str.2006.09.013 17161365

[33] Groemping Y., Lapouge K., Smerdon S. J., and Rittinger K. (2003) Molecular basis of phosphorylation-induced activation of the NADPH oxidase. Cell 113, 343–355 10.1016/S0092-8674(03)00314-3 12732142

[34] Yuzawa S., Suzuki N. N., Fujioka Y., Ogura K., Sumimoto H., and Inagaki F. (2004) A molecular mechanism for autoinhibition of the tandem SH3 domains of p47phox, the regulatory subunit of the phagocyte NADPH oxidase. Genes Cells 9, 443–456 10.1111/j.1356-9597.2004.00733.x 15147273

[35] Kishan K. V., Scita G., Wong W. T., Di Fiore P. P., and Newcomer M. E. (1997) The SH3 domain of Eps8 exists as a novel intertwined dimer. Nat. Struct. Biol. 4, 739–743 10.1038/nsb0997-739 9303002

[36] Bacarizo J., Martinez-Rodriguez S., Martin-Garcia J. M., Andujar-Sanchez M., Ortiz-Salmeron E., Neira J. L., and Camara-Artigas A. (2014) Electrostatic effects in the folding of the SH3 domain of the c-Src tyrosine kinase: pH-dependence in 3D-domain swapping and amyloid formation. PLoS ONE 9, e113224 10.1371/journal.pone.0113224 25490095 PMC4260792

[37] Cámara-Artigas A., Martín-García J. M., Morel B., Ruiz-Sanz J., and Luque I. (2009) Intertwined dimeric structure for the SH3 domain of the c-Src tyrosine kinase induced by polyethylene glycol binding. FEBS Lett. 583, 749–753 10.1016/j.febslet.2009.01.036 19185573

[38] Matsumura Y., Shinjo M., Matsui T., Ichimura K., Song J., and Kihara H. (2013) Structural study of hNck2 SH3 domain protein in solution by circular dichroism and X-ray solution scattering. Biophys. Chem. 175, 39–46 10.1016/j.bpc.2013.02.005 23524290 PMC3925460

[39] Ding F., Dokholyan N. V., Buldyrev S. V., Stanley H. E., and Shakhnovich E. I. (2002) Molecular dynamics simulation of the SH3 domain aggregation suggests a generic amyloidogenesis mechanism. J. Mol. Biol. 324, 851–857 10.1016/S0022-2836(02)01112-9 12460582

[40] Bennett M. J., Sawaya M. R., and Eisenberg D. (2006) Deposition diseases and 3D domain swapping. Structure 14, 811–824 10.1016/j.str.2006.03.011 16698543

[41] Frese S., Schubert W. D., Findeis A. C., Marquardt T., Roske Y. S., Stradal T. E., and Heinz D. W. (2006) The phosphotyrosine peptide binding specificity of Nck1 and Nck2 Src homology 2 domains. J. Biol. Chem. 281, 18236–18245 10.1074/jbc.M512917200 16636066

[42] Banjade S., Wu Q., Mittal A., Peeples W. B., Pappu R. V., and Rosen M. K. (2015) Conserved interdomain linker promotes phase separation of the multivalent adaptor protein Nck. Proc. Natl. Acad. Sci. U.S.A. 112, E6426–E6435 10.1073/pnas.1508778112 26553976 PMC4664304

[43] Huculeci R., Kieken F., Garcia-Pino A., Buts L., van Nuland N., and Lenaerts T. (2017) Structural characterization of monomeric/dimeric state of p59(fyn) SH2 domain. Methods Mol. Biol. 1555, 257–267 10.1007/978-1-4939-6762-9_14 28092037

[44] Benfield A. P., Whiddon B. B., Clements J. H., and Martin S. F. (2007) Structural and energetic aspects of Grb2-SH2 domain-swapping. Arch. Biochem. Biophys. 462, 47–53 10.1016/j.abb.2007.03.010 17466257 PMC1947945

[45] Hosoe Y., Numoto N., Inaba S., Ogawa S., Morii H., Abe R., Ito N., and Oda M. (2019) Structural and functional properties of Grb2 SH2 dimer in CD28 binding. Biophys. Physicobiol. 16, 80–88 10.2142/biophysico.16.0_80 30923665 PMC6435016

[46] Saint-Jean A. P., Phillips K. R., Creighton D. J., and Stone M. J. (1998) Active monomeric and dimeric forms of *Pseudomonas putida* glyoxalase I: evidence for 3D domain swapping. Biochemistry 37, 10345–10353 10.1021/bi980868q 9671502

[47] Schymkowitz J. W., Rousseau F., Wilkinson H. R., Friedler A., and Itzhaki L. S. (2001) Observation of signal transduction in three-dimensional domain swapping. Nat. Struct. Biol. 8, 888–892 10.1038/nsb1001-888 11573096

[48] Bennett M. J., Schlunegger M. P., and Eisenberg D. (1995) 3D domain swapping: a mechanism for oligomer assembly. Protein Sci. 4, 2455–2468 10.1002/pro.5560041202 8580836 PMC2143041

[49] Baker M. A., Hynson R. M., Ganuelas L. A., Mohammadi N. S., Liew C. W., Rey A. A., Duff A. P., Whitten A. E., Jeffries C. M., Delalez N. J., Morimoto Y. V., Stock D., Armitage J. P., Turberfield A. J., Namba K., et al (2016) Domain-swap polymerization drives the self-assembly of the bacterial flagellar motor. Nat. Struct. Mol. Biol. 23, 197–203 10.1038/nsmb.3172 26854663

[50] Concordet J. P., and Haeussler M. (2018) CRISPOR: intuitive guide selection for CRISPR/Cas9 genome editing experiments and screens. Nucleic Acids Res. 46, W242–W245 10.1093/nar/gky354 29762716 PMC6030908

[51] Lucas L. H., and Larive C. K. (2004) Measuring ligand-protein binding using NMR diffusion experiments. Concepts Magn. Reson. A 20A, 24–41 10.1002/cmr.a.10094

[52] Kabsch W. (2010) XDS. Acta Crystallogr. D Biol. Crystallogr. 66, 125–132 10.1107/S0907444909047337 20124692 PMC2815665

[53] Karplus P. A., and Diederichs K. (2012) Linking crystallographic model and data quality. Science 336, 1030–1033 10.1126/science.1218231 22628654 PMC3457925

[54] Diederichs K., and Karplus P. A. (2013) Better models by discarding data? Acta Crystallogr. D Biol. Crystallogr. 69, 1215–1222 10.1107/S0907444913001121 23793147 PMC3689524

[55] Winn M. D., Ballard C. C., Cowtan K. D., Dodson E. J., Emsley P., Evans P. R., Keegan R. M., Krissinel E. B., Leslie A. G., McCoy A., McNicholas S. J., Murshudov G. N., Pannu N. S., Potterton E. A., Powell H. R., et al (2011) Overview of the CCP4 suite and current developments. Acta Crystallogr. D Biol. Crystallogr. 67, 235–242 10.1107/S0907444910045749 21460441 PMC3069738

[56] McCoy A. J., Grosse-Kunstleve R. W., Adams P. D., Winn M. D., Storoni L. C., and Read R. J. (2007) Phaser crystallographic software. J. Appl. Crystallogr. 40, 658–674 10.1107/S0021889807021206 19461840 PMC2483472

[57] Emsley P., Lohkamp B., Scott W. G., and Cowtan K. (2010) Features and development of Coot. Acta Crystallogr. D Biol. Crystallogr. 66, 486–501 10.1107/S0907444910007493 20383002 PMC2852313

[58] Murshudov G. N., Skubák P., Lebedev A. A., Pannu N. S., Steiner R. A., Nicholls R. A., Winn M. D., Long F., and Vagin A. A. (2011) REFMAC5 for the refinement of macromolecular crystal structures. Acta Crystallogr. D Biol. Crystallogr. 67, 355–367 10.1107/S0907444911001314 21460454 PMC3069751

[59] Adams P. D., Afonine P. V., Bunkóczi G., Chen V. B., Davis I. W., Echols N., Headd J. J., Hung L. W., Kapral G. J., Grosse-Kunstleve R. W., McCoy A. J., Moriarty N. W., Oeffner R., Read R. J., Richardson D. C., et al (2010) PHENIX: a comprehensive Python-based system for macromolecular structure solution. Acta Crystallogr. D Biol. Crystallogr. 66, 213–221 10.1107/S0907444909052925 20124702 PMC2815670

[60] Vistica J., Dam J., Balbo A., Yikilmaz E., Mariuzza R. A., Rouault T. A., and Schuck P. (2004) Sedimentation equilibrium analysis of protein interactions with global implicit mass conservation constraints and systematic noise decomposition. Anal. Biochem. 326, 234–256 10.1016/j.ab.2003.12.014 15003564

[61] Brautigam C. A. (2015) Calculations and publication-quality illustrations for analytical ultracentrifugation data. Methods Enzymol. 562, 109–133 10.1016/bs.mie.2015.05.001 26412649

