# Supplementary material for: Small molecule AX-024 reduces T cell proliferation independently of CD3ϵ/Nck1 interaction, which is governed by a domain swap in the Nck1-SH3.1 domain
Source: J Biol Chem. 2020 Apr 21;295(23):7849–64. doi: 10.1074/jbc.RA120.012788 (PMC7278359; doi:10.1074/jbc.RA120.012788)
Supplement: Supporting Information [file supp_295_23_7849__index.html]

Small molecule AX-024 reduces T cell proliferation independently of CD3&epsilon;-Nck1 interaction, which is governed by a domain-swap in the Nck1-SH3.1 domain — Conformational variety in the Nck1 SH3.1 domain — Small molecule AX-024 reduces T cell proliferation independently of CD3ϵ/Nck1 interaction, which is governed by a domain swap in the Nck1-SH3.1 domain — Conformational variety in the Nck1 SH3.1 domain — Supporting Information 

# Small molecule AX-024 reduces T cell proliferation independently of CD3ϵ/Nck1 interaction, which is governed by a domain swap in the Nck1-SH3.1 domain

## Supporting Information

- Supporting Information (to be published online) - Figures and Tables
