# Supplementary material for: Small molecule AX-024 reduces T cell proliferation independently of CD3ϵ/Nck1 interaction, which is governed by a domain swap in the Nck1-SH3.1 domain
Source: J Biol Chem. 2020 Apr 21;295(23):7849–64. doi: 10.1074/jbc.RA120.012788 (PMC7278359; doi:10.1074/jbc.RA120.012788)
Supplement: Supporting Information [file supp_RA120.012788_158427_2_supp_511874_q93hr0.pdf]

TABLES

Supplemental Table 1: SPR peptide titrations

Replicate #	K_d ± error (μM)	RU_{max} (RU)	Average K_d (μM)
1	8.5 ± 0.4	44.8	
2	8.4 ± 0.3	42.6	
3	8.7 ± 0.3	40.0	8.3 ± 0.1
4	8.0 ± 0.4	38.1	
5	7.8 ± 0.5	36.4	

Repeated re-generation of the sensor surface between individual titrations leads to a decrease in maximal response RU_{max}, which does not affect the data quality.

Supplemental Table 2: Targets whose activity is inhibited > 50% by 10 μM AX-024.

**Target**	**% inhibition**
Histamine H3	95.1
Muscarinic M1	95.0
Dopamine D1	85.3
Muscarinic M2	81.3
5-HT2B	80.1
α 1A adrenoreceptor	79.3
Ca^{2+} channel (CaV1.2) (L, diltiazem site)	66.3

Single point measurements at 10 μM for 50 targets in a selectivity panel. Targets inhibited by AX-024 are human receptors, except for the Ca^{2+} channel, which is from rat. A radioactive antagonist ligand was used for the detection. The tests were conducted by Eurofins (https://www.eurofinsdiscoveryservices.com).

Supplemental Table 3: Crystallization conditions

PDB-ID	precipitant	construct	state
5QU1	0.1 M Bis-Tris/HCl pH 6.5, 2 M $(NH_4)_2SO_4$	4-59	monomer
5QU2	0.1 M Bis-Tris/HCl pH 6.5, 2 M $(NH_4)_2SO_4$	4-59	monomer
5QU3	0.1M Tris/HCl pH 8.0, 0.15 M NaCl, 8% PEG 8000	4-59	dimer
5QU4	0.1 M KSCN, 30% PEG-MME 2K	1-61	dimer
5QU5	0.1 M Bis-Tris/HCl pH 5.5, 2 M NaCl, 25% PEG 3350	1-61	dimer
5QU6	0.1 M Bis-Tris/HCl pH 6.5, 2% tacsimate pH 6.0, 20% PEG 3350	1-61	dimer
5QUA	0.1 M Bis-Tris/HCl pH 6.5, 2 M $(NH_4)_2SO_4$	4-59	dimer
5QU7	8% tacsimate pH 5, 20% PEG 3350	4-59	dimer
5QU8	60% tacsimate pH 7.0	1-61	dimer [*)]

Tacsimate contains 1.8305 M malonic acid, 0.25 M ammonium citrate tribasic, 0.12 M succinic acid, 0.3 M DL-malic acid, 0.4 M NaOAc trihydrate, 0.5 M sodium formate, and 0.16 M ammonium tartrate dibasic, titrated with NaOH. [*)] A single polypeptide chain is present in the asymmetric unit. The swapped dimer is created by crystallographic symmetry.

Supplemental Table 4: X-ray data collection and refinement statistics

**Structure**	**5QU1**	**5QU2, Nck1-SH3.1/CD3ε**	**5QU3**	**5QU4**	**5QU5**
Space group	P1	$I2_12_12_1$	$P2_1$	$P2_12_12_1$	$P2_12_12_1$
Copies / ASU	2	2	2	4	2
Cell a,b,c (Å)	23.9, 28.4, 38.6	50.7, 63.3, 99.7	29.5, 55.7, 39.9	52.2, 57.5, 85.4	40.2, 55.2, 55.2
Cell α, β, γ (°)	87.2, 86.5, 67.0	90, 90, 90	90, 98.7, 90	90, 90, 90	90, 90, 90
V_M ($Å^3$/Da) / Solv. Cont. (%)	2.0 / 37.0	2.8 / 56.0	2.6 / 52.8	2.6 / 51.9	2.4 / 47.9
Resolution (Å) [1)]	38.5 (1.22-1.09)	49.8 (1.13-1.05)	32.2 (1.10-1.02)	85.4 (1.17-1.05)	32.5 (1.28-1.12)
100% criterion (Å) [2)]	1.41	1.26	1.16	1.19	1.35
Total # of reflections	41665 (2120)	287022 (14265)	215918 (10331)	563454 (25701)	198597 (7582)
Multiplicity	2.0 (2.0)	6.5 (6.5)	4.9 (4.7)	7.0 (6.4)	7.0 (5.3)
Completeness [3)] (%)	79 (29) / 53 (9.4)	88 (64) / 58 (15)	89 (52) / 68 (18)	95 (80) / 68 (13)	89 (46) / 60 (9)
R_{meas} [4)]	10.4	6.3	5.4	6.3	6.1
$CC_{1/2}$ [4)]	0.99 (0.67)	1.0 (0.51)	1.0 (0.43)	1.0 (0.75)	1.0 (0.64)
I/σ(I)	6.5 (1.8)	14.5 (1.6)	12.6 (1.3)	12.8 (1.8)	14 (1.8)
Wilson B-value ($Å^2$)	4.9	9.8	8.4	9.9	11.3
Refinement	REFMAC5	REFMAC5	REFMAC5	REFMAC5	REFMAC5
R_{cryst} (%) / R_{free} (%) [5)]	16.1 / 21.4	16.8 / 20.5	16.5 / 18.9	15.5 / 18.6	15.5 / 20.8
Residues / H_2O	112 / 135	130 / 173	113 / 206	228 / 309	118 / 158
rmsd bonds/angles	0.013/1.63	0.015/1.94	0.013/1.69	0.024/2.18	0.022/2.02
DPI / phase error (Å / °) [6)]	0.07 / 32	0.05 / 31	0.04 / 27	0.04 / 24	0.06 / 36
Ramachandran plot [7)]	98.2/0	97.5/0	100/0	98.2/0	98.3/0
Molprobity / Clashscore [8)]	1.04 / 2.5	1.18 / 3.0	1.39 / 4.6	1.81 / 6.8	1.19 / 1.0
<B> ($Å^2$) protein / H_2O	8.3±7.0 / 18.2±8.5	14.4±7.4 / 28.0±8.5	12.5±6.3 / 30.3±10.6	17.7±11.1 / 27.6±8.2	19.5±11.6 / 31.8±8.8

Supplemental Table 4 (cont.): X-ray data collection and refinement statistics

Structure	**5QU6**	**5QUA**	**5QU7**	**5QU8**
Space group	P1	$C222_1$	$P2_12_12_1$	$P2_12_12$
Copies / ASU	28	2	2	1
Cell a,b,c (Å)	49.1, 92.1, 114.7	53.6, 85.2, 56.2	40.1, 48.9, 55.1	40.1, 55.2, 27.0
Cell α, β, γ (°)	103.0, 91.6, 88.6	90, 90, 90	90, 90, 90	90, 90, 90
V_M ($Å^3$/Da) / Solv. Cont. (%)	3.0 / 58.6	2.6 / 52.8	2.2 / 42.9	2.3 / 47.5
Resolution (Å) [1)]	55.9 (2.04-1.82)	35.3 (1.64-1.52)	40.1 (1.42-1.27)	27.6 (1.06-0.93)
100% criterion (Å) [2)]	1.92	1.68	1.43	1.22
Total # of reflections	220258 (11957)	97838 (4503)	148941 (4928)	113359 (4951)
Multiplicity	2.0 (2.2)	6.3 (5.8)	7.1 (4.7)	5.8 (5.1)
Completeness (%) [2)]	88 (52) / 63 (11)	94 (58) / 77 (20)	93 (57) / 73 (14)	90.4 (67) / 47.4 (7)
R_{meas} [4)]	5.2	2.6	8.1	5.7
$CC_{1/2}$ [4)]	1.0 (0.67)	1.0 (0.47)	1.0 (0.66)	1.0 (0.54)
I/σ(I)	10.5 (1.7)	29.2 (1.1)	12.3 (1.5)	12.9 (1.5)
Wilson B-value ($Å^2$)	29.6	34.6	14.9	8.5
Refinement	PHENIX	REFMAC5	REFMAC5	REFMAC5
R_{cryst} (%) / R_{free} (%) 5)	21.1 / 26.3	20.1 / 22.7	17.3 / 21.9	15.1 / 18.2
Residues / H_2O	1546 / 213	112 / 62	114 / 105	58 / 66
R.m.s.d. bonds/angles	0.008/0.91	0.015/1.91	0.013/1.69	0.016/1.86
DPI / phase error (Å/°) [6)]	0.20 / 39.3	0.10 / 42	0.07 / 31	0.04 / 34
Ramachandran plot [7)]	97.0/0.1	93.5/1.9	98.2/0	98.2/0
Molprobity / Clashscore [8)]	2.03 / 6.6	2.08 / 5.8	1.63 / 4.4	1.16 / 3.7
<B> ($Å^2$) protein, H_2O	58.2±26.6 / 40.8±9.7	34.0±14.6 / 42.8±7.8	21.9±11.8 / 34.4±8.5	17.7±14.4 / 23.2±10.3

[1)] Values in parentheses correspond to the highest resolution shell. [2)] The 100% criterion was calculated using SFTOOLS (55) and represents the resolution in Å of a 100% complete hypothetical data set with the same number of reflections as the measured data. [3)] Completeness is given for ellipsoidal / spherical cutoff as defined by STARANISO (staraniso.globalphasing.org). [4)] R-values and $CC_{1/2}$ are defined in (62) and (53), respectively, and were calculated with PHENIX (63). [5)] $R_{cryst} = \Sigma||F_o|-|F_c||/\Sigma|F_o|$, where F_o and F_c are the structure factor amplitudes from the data and the model, respectively. R_{free} is R_{cryst} with 5% of test set structure factors. [6)] Cruickshank diffraction-component precision index based on R-value (64). [7)] Calculated using PHENIX (63). Numbers reflect the percentage of amino acid residues in the favored and disallowed regions, respectively. [8)] Molprobity score should approach the high-resolution limit (65). Clashscore is defined as the number of unfavorable all-atom steric overlaps ≥ 0.4Å per 1000 atoms (66).

Supplemental Figures

Suppl. Fig. 1

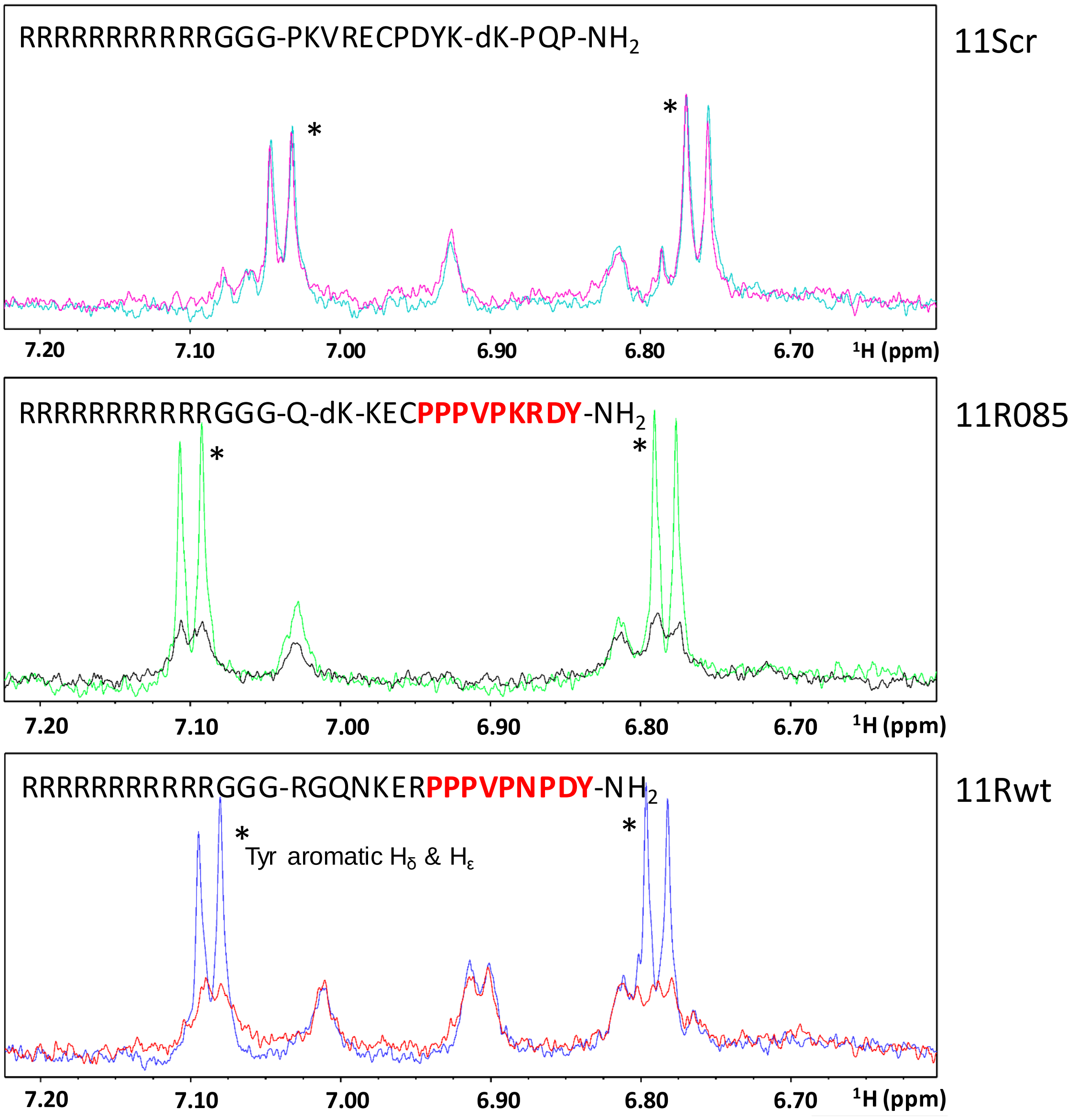

The three panels each show a superposition of two 1D spectra, one of the peptide indicated and another with Nck1-SH3.1 (residues 4-59) added. The top panel is a control using a scrambled peptide (11RScr) of the same composition as 11R085 but that does not bind to Nck1 (cyan: peptide alone, magenta: with Nck1-SH3.1 added). The middle and bottom panels show peptides that do bind to Nck1-SH3.1. The SH3.1-domain binding motif is indicated in red in the peptide sequence. “dK” denotes the other enantiomer of lysine at this position. Aromatic signals of the N-terminal tyrosine (see asterisks) are significantly broadened when the peptides bind to the SH3.1 domain.

Suppl. Fig. 2

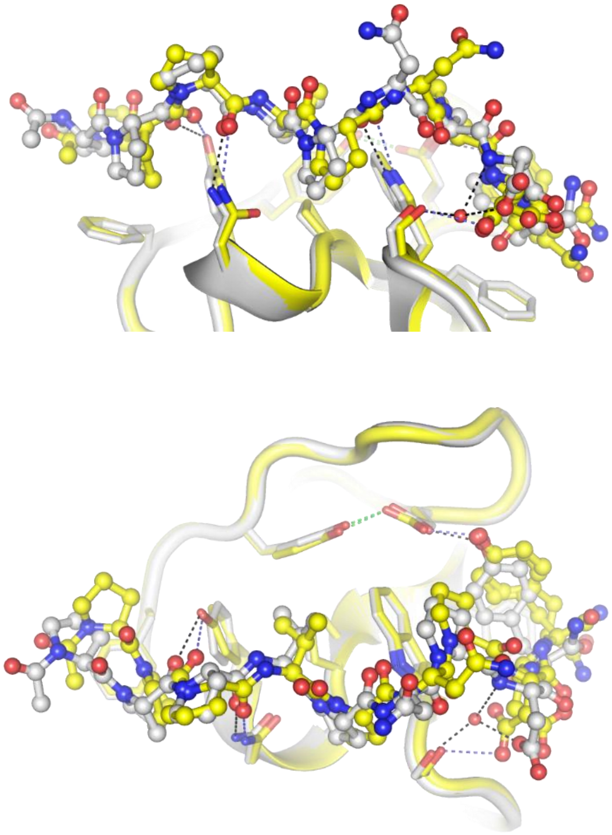

Superposition of the two peptide/SH3.1 complexes in the asymmetric unit. Peptides are shown in ball-and-stick representation. The top panel has the same orientation as Figure 7B, the bottom panel is rotated by ca. 90° about the horizontal axis, as in Figure 7C. A slight lateral shift is apparent for the peptides, indicating some flexibility when bound to SH3.1. In the second complex (yellow), the C-terminal peptide residues Asp187 and Tyr188 adopt two alternative conformations. In one of the conformations, Asp187 replaces the water molecule found in the first complex (grey) and hydrogen bonds directly to Ser37. Both conformations of the C-terminal peptide Tyr188 sidechain bind to Glu20 of SH3.1. The intra-SH3.1 hydrogen bond between the side-chains of Tyr13 and Glu20 is drawn in green. It is present in both, apo- and peptide-bound structures.

Suppl. Fig. 3

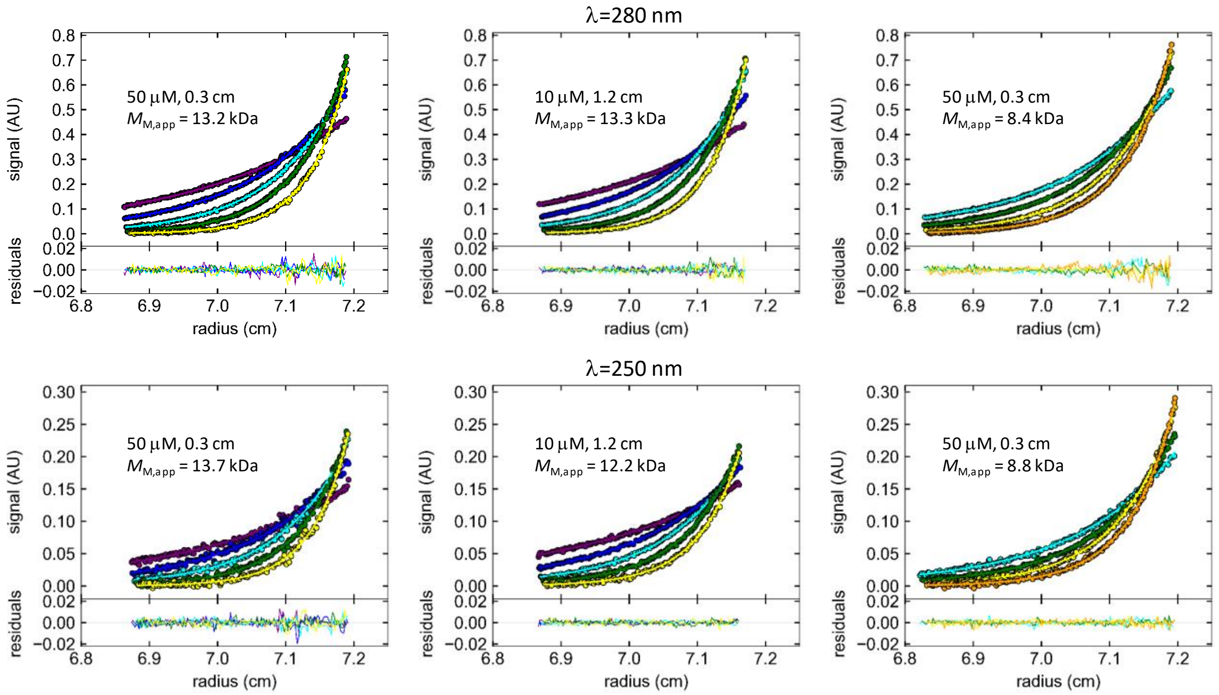

The top three panels were measured at 280 nm, the bottom three at 250 nm. The sample concentrations, path lengths, and apparent molecular masses are indicated. The right-most panels show the results from a heat-treated dimeric Nck1-SH3.1 sample (24 h at 42°C). The top left and top right panels are identical to Fig. 11 in the main text.
